# A gene regulatory network combining Pax3/7, Sox10 and Mitf generates diverse pigment cell types in medaka and zebrafish

**DOI:** 10.1242/dev.202114

**Published:** 2023-10-12

**Authors:** Motohiro Miyadai, Hiroyuki Takada, Akiko Shiraishi, Tetsuaki Kimura, Ikuko Watakabe, Hikaru Kobayashi, Yusuke Nagao, Kiyoshi Naruse, Shin-ichi Higashijima, Takashi Shimizu, Robert N. Kelsh, Masahiko Hibi, Hisashi Hashimoto

**Affiliations:** ^1^ Laboratory of Biological Science, Division of Natural Science, Graduate School of Science, Nagoya University, Nagoya 464-8602, Japan; ^2^ Laboratory of Bioresources, National Institute for Basic Biology, Okazaki 444-8585, Japan; ^3^ National Institutes of Natural Sciences, Exploratory Research Center on Life and Living Systems, National Institute for Basic Biology, Okazaki 444-8787, Japan; ^4^ Department of Life Sciences, University of Bath, Bath BA2 7AY, UK

**Keywords:** Paired-type homeobox, Chromatophore, Melanocyte, Pigmentation, CRISPR/Cas9

## Abstract

Neural crest cells generate numerous derivatives, including pigment cells, and are a model for studying how fate specification from multipotent progenitors is controlled. In mammals, the core gene regulatory network for melanocytes (their only pigment cell type) contains three transcription factors, Sox10, Pax3 and Mitf, with the latter considered a master regulator of melanocyte development. In teleosts, which have three to four pigment cell types (melanophores, iridophores and xanthophores, plus leucophores e.g. in medaka), gene regulatory networks governing fate specification are poorly understood, although Mitf function is considered conserved. Here, we show that the regulatory relationships between Sox10, Pax3 and Mitf are conserved in zebrafish, but the role for Mitf is more complex than previously emphasized, affecting xanthophore development too. Similarly, medaka Mitf is necessary for melanophore, xanthophore and leucophore formation. Furthermore, expression patterns and mutant phenotypes of *pax3* and *pax7* suggest that Pax3 and Pax7 act sequentially, activating *mitf* expression. Pax7 modulates Mitf function, driving co-expressing cells to differentiate as xanthophores and leucophores rather than melanophores. We propose that pigment cell fate specification should be considered to result from the combinatorial activity of Mitf with other transcription factors.

## Teaser

**Summary:** Unique and sequential functions of Pax3 and Pax7, in combination with Mitf, allow the diversification of pigment cell types in teleost.

## INTRODUCTION

All body pigment cells in vertebrates are considered to derive from neural crest cells (NCCs), a fascinating population of multipotent stem cells (Le Douarin and Kalcheim, 1999; Tang and Bronner, 2020). These cells form an interesting class of NCC derivatives, in part because traditionally all pigment cell types have been believed to develop from partially restricted NCC-derived progenitor cells, so called chromatoblasts (Bagnara et al., 1979; Schartl et al., 2016), although we have recently challenged that view, showing that neural crest cells appear to retain much broader multipotency, even in differentiating pigment cells (Kelsh et al., 2021; Subkhankulova et al., 2023). Regardless of their exact nature, these multipotent progenitor cells undergo a process of fate specification to one or more cell types, before differentiation and eventual commitment to individual specialized cell types. This process is regulated by a complex gene regulatory network (GRN) organized around a core set of transcription factors, which ultimately directs cells to differentiate, stably expressing a transcriptome appropriate to the specific cell type.

Mammals and birds have only one type of pigment cell, called the melanocyte (Sommer, 2011; Wegner, 2005). The core GRN that controls melanocyte differentiation consists of three transcription factors: the Sry-related HMG-type transcription factor Sox10, the paired-type homeodomain-containing Pax transcription factor Pax3 and the basic helix-loop-helix (bHLH) transcription factor Mitf (microphthalmia-associated transcription factor). Sox10 and Pax3 cooperatively promote melanocyte development through activating expression of Mitf, which in turn drives melanin-synthesizing enzymes, e.g. dopachrome tautomerase (Dct) (Jiao et al., 2004; Lang et al., 2005; Ludwig et al., 2004; Tsukamoto et al., 1992). Mutations in *SOX10*, *PAX3* or *MITF* similarly cause Waardenburg syndrome in humans (Bondurand et al., 2000; Potterf et al., 2000; Tassabehji et al., 1992), which manifests as patchy pigmentation due to (partial) loss of melanocytes in the skin. Mitf is considered to be a master regulator of melanocytes in mammals as *MITF/Mitf* mutations result in absence of melanocytes, and MITF target genes are numerous and include at least the majority of melanocyte markers (Arnheiter, 2010; Goding and Arnheiter, 2019; Hemesath et al., 1994; Kawakami and Fisher, 2017; Opdecamp et al., 1997; Steingrimsson et al., 1994).

In contrast to mammals and birds, other vertebrates basically have three different types of pigment cell: the melanophore (equivalent to the mammalian melanocyte), the iridophore (iridescent cell) and the xanthophore (yellow to red pigment cell). A few species of teleosts, including the Japanese endemic fish medaka, have a fourth type, the leucophore (white cell), but these are absent from the zebrafish except for a few cells (which may or may not be homologous) in adults (Hashimoto, 2021; Lewis et al., 2019). A more complex GRN must be required to generate this diversity of pigment cell types. Here, we address this question of defining the GRN that achieves fate specification and differentiation of the multiple pigment cells in ectothermic vertebrates. We test the hypothesis that the core GRN is conserved in both endothermic and ectothermic vertebrates, but achieves added complexity through some additional components specific to ectothermic vertebrates (secondarily lost in the endothermic taxa). Among the three core transcription factors, Sox10 and Mitf have been shown to have highly conserved roles in melanocyte/melanophore development, whereas the function of Pax3 is still poorly understood in other vertebrate species (Greenhill et al., 2011; Hou et al., 2006).

Sox10 plays central roles in all non-ectomesenchymal fates of NCCs, in both fish and mammals, with its loss resulting in absence or severe depletion of all NCC-derived neuronal, glial and pigment cell types (Britsch et al., 2001; Dutton et al., 2001; Kelsh and Eisen, 2000; Nagao et al., 2018; Southard-Smith et al., 1998). Evidence to date suggests that development of each of these derivatives fails at the earliest stages of specification from the NCCs (Dutton et al., 2001; Elworthy et al., 2003, 2005; Greenhill et al., 2011; Kelsh, 2006; Lopes et al., 2008; Petratou et al., 2021, 2018). Thus, in zebrafish and medaka, loss of Sox10 function results in an almost-complete lack of melanophores (except for the brain-derived pigmented retinal epithelium), xanthophores and iridophores (Tsunogai et al., 2021), and in mice it results in complete lack of melanocytes (Britsch et al., 2001; Southard-Smith et al., 1998).

As in mammals, the zebrafish MITF homologue, Mitfa, plays a crucial role in melanophore development as zebrafish *mitfa^w2/w2^* mutants (also known as *nacre*) lack melanophores throughout life (Lister et al., 1999). A crucial role in melanophore fate specification and differentiation is indicated by the extensive absence of melanophore markers in zebrafish *mitfa* mutants and by the role of Mitfa in directly driving *dct* transcription and melanin pigmentation in the melanophore lineage in zebrafish (Greenhill et al., 2011; Lister et al., 1999), as in mice (Jiao et al., 2004; Ludwig et al., 2004; Tsukamoto et al., 1992). Furthermore, expression of *mitfa* in the NCCs of zebrafish *sox10* mutants is sufficient to rescue full melanophore differentiation (Elworthy et al., 2003), consistent with the master regulator model proposed for mammalian MITF. However, although xanthophores are formed in zebrafish *mitfa^w2/w2^* mutants, their development has been noted as abnormal, suggesting that Mitfa may have a partially redundant role in xanthophore formation (Lister et al., 1999). Nevertheless, the exact role of Mitfa in xanthophore development remains unknown.

Pax3 functions as a cooperative partner of Sox10, and thus is also required for melanocyte specification in mammals (Kubic et al., 2008). *Pax3* mutations result in loss of coat colour in the *Splotch* mutant mice (Conway et al., 1997; Tremblay et al., 1995), and also in splashed white horses (Hauswirth et al., 2012). In teleosts, however, the role of Pax3 is not clear. An antisense morpholino-mediated knockdown study in zebrafish showed that loss of Pax3 resulted in defective specification of xanthophores, but not of melanophores or iridophores (Minchin and Hughes, 2008).

Pax7 is closely related to Pax3 and has been suggested to have similar activity to Pax3. These two transcription factors play important roles in a variety of stem cells, including the NCCs, such as regulating fate decision and differentiation (Buckingham and Relaix, 2015; Nord et al., 2022; Relaix et al., 2004). The similarity is considered to reflect their emergence from a common ancestral gene through duplication (Robson et al., 2006). *Pax7* knockdown by morpholino in the chick indicates that Pax7 is important for induction of the neural crest from the ectoderm, but not specifically required for melanocyte differentiation (Basch et al., 2006). To date, no *PAX7-*associated pigmentation disorders have been reported in humans and mice, although *Pax7*-expressing cells give rise to NCCs (Murdoch et al., 2012). Thus, Pax7 does not appear to play a major role in mammalian or avian melanocyte development.

Meanwhile, in teleosts, Pax7 seems to be involved in the GRN for pigment cell development. Zebrafish *pax7a; pax7b* double-homozygous mutants exhibit complete absence of xanthophores, but not of melanophores nor iridophores (Nord et al., 2016), suggesting an essential and specific role of Pax7 in xanthophore formation. However, in medaka, in which functional Pax7b has been evolutionarily lost, *pax7a* homozygous mutants exhibit a complete absence of xanthophores and leucophores. As a consequence, xanthophores and leucophores have been proposed to share a bipotent progenitor, specification of which requires Pax7a function in medaka (Kimura et al., 2014; Nagao et al., 2014, 2018). Although that conclusion may need revision in the context of our recent zebrafish observations (Kelsh et al., 2021; Subkhankulova et al., 2023), it remains clear that Pax7 function is necessary for both xanthophore and leucophore development.

Here, we used a comparative genetic approach to assess the roles for these key pigment cell transcription factors. We addressed the following key questions: (1) Is Pax3 one of the core transcription factors in the melanophore GRN in medaka and zebrafish?; (2) Do Pax3 and Pax7 control specification of other pigment cell types?; and (3) Does Mitfa have a role in the development of other pigment cell types in medaka? Our results suggest that Sox10, Pax3 and Mitf have a conserved role as components of the core GRN driving melanophore fate, but that they also work alongside Pax7 to promote xanthophore and leucophore fates in teleosts.

## RESULTS

### Two paralogous *pax3* genes, *pax3a* and *pax3b*, are identified in medaka and zebrafish genomes

Vertebrate *pax3* and *pax7* are descendants of a common ancestral gene (named *pax37* in ascidians), resulting from the second-round whole genome duplication in vertebrate evolution (Kusakabe and Kuratani, 2007; Wada et al., 1996). In teleosts, two paralogues for each of *pax3* and *pax7* were generated as a result of another round of whole-genome duplication (Amores et al., 1998; Postlethwait et al., 2004). Medaka and zebrafish have *pax3a*, *pax3b*, *pax7a* and *pax7b* in their genomes, although medaka *pax7b* lacks three exons (exons 6-8) and appears to be a non-functional pseudogene (see Fig. S1).

### *pax3* is expressed in neural crest cells prior to *pax7*

**Fig. 1.**
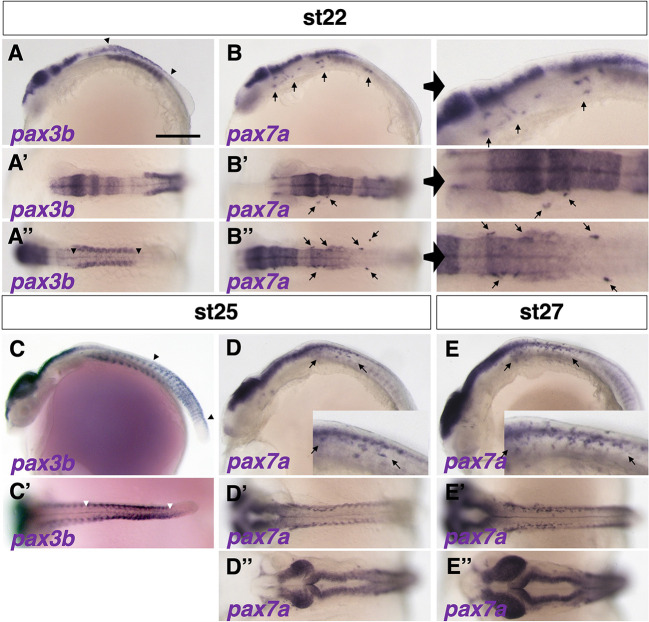
**Expression patterns of *pax3* and *pax7* in medaka.** (A-E″) Expression of *pax3b* mRNA was detected in NCCs at stage 22 (9 somite embryo) (A-A″, between arrowheads), whereas *pax7a* expression was observed in migrating NCCs anteroventral to the *pax3b*-expressing premigratory NCCs (B-B″; panels on the right show enlarged views; *pax7a*-expressing cells are indicated by arrows). The *pax3b* expression had shifted posteriorly at stage 25 (18-19 somite embryo) (C,C′, between arrowheads) and became undetectable by stage 27 (24 somite embryo) (not shown). *pax7a* mRNA was continuously observed in migrating NCCs from stage 25 to stage 27 (indicated by arrows; see also Fig. S2). *pax3b* and *pax7a* mRNAs were expressed in similar regions of neural tissue and somites (A-E). Expression of *pax3b* was observed in several anterior somites at stage 22 (A), shifted to posterior somites at stage 25 (C; see also Fig. S2), and appeared to precede that of *pax7a* in somites (B,D). The time windows of *pax3b* and *pax7a* expression overlapped during stages 22-25. (A-E) Lateral views. (A′-E′,A″,B″,D″,E″) Dorsal views. Images are representative of more than ten embryos. Scale bar: 250 µm.

We observed expression of only *pax3b* and *pax7a* mRNAs in medaka NCCs. *In situ* hybridization analyses showed that *pax3b*-expressing cells were present in dorsal parts of the midbrain, hindbrain and spinal cord, dorsal somites and premigratory NCCs at early somite stages (Fig. 1A-A″). In contrast, *pax7a* expression was detected in both premigratory and laterally migrating NCCs (Fig. 1B-B″). At mid-somite stages, *pax3b*-expressing NCCs were seen in the posterior premigratory region, but not in the lateral migratory region (Fig. 1C,C′), whereas *pax7a*-expressing NCCs were observed in both premigratory and migrating regions (Fig. 1D-D″). By late somite stages, *pax3b* expression was no longer detectable in the NCCs, but *pax7a* expression was still detected in migratory NCCs (Fig. 1E-E″) and maintained until almost the hatching stage (Watakabe et al., 2018). These results indicate that expression of *pax3b* in medaka is transient, restricted to premigratory NCCs, and precedes that of *pax7a*, which is maintained into migrating NCCs. These expression patterns are similar to those previously reported for zebrafish *pax3* (*pax3a* and *pax3b*) and *pax7* (*pax7a* and *pax7b*) (Minchin and Hughes, 2008).

The above results were confirmed by observing fluorescently labelled *pax3b*- and *pax7a*-expressing cells in a newly established stable transgenic medaka Tg*(pax3b-hs:GFP)* and previously reported Tg*(pax7a-hs:GFP)* lines (Fig. S2A) (Watakabe et al., 2018). *pax7a*-GFP remained detectable in NCCs until a late somite stage (Fig. S2D,F,H), much longer than the *pax3b*-GFP (Fig. S2B,C,E,G). We also tested whether the expression of *pax3b* and *pax7a* overlapped, by using Tg(*pax3b*-*hs*:*GFP*) crossed with the previously reported Tg*BAC*(*pax7a*:*DsRed*) (Nagao et al., 2018). A portion, but not all, of the cells positive for *pax3b*-GFP were also positive for *pax7a-*DsRed (Fig. S2I-K), indicating that these cells express both Pax3b and Pax7a. We observed *pax3b*-GFP-positive but *pax7a*-DsRed-negative cells, which may be cells that have not yet reached the state at which they are ready to express *pax7a*, but which may indicate that *pax3b*-expressing cells give rise to pigment cell populations other than *pax7a*-expressing xanthophore/leucophore progenitors.

### Loss of *pax3* function results in delayed formation of xanthophores in zebrafish and medaka, and also of leucophores in medaka

To investigate the role of *pax3* and *pax7*, we generated mutations in *pax3a* and *pax3b* in medaka (Fig. S1) and *pax3a*, *pax3b*, *pax7a* and *pax7b* in zebrafish using transcription activator-like effector nucleases (TALENs) or CRISPR/Cas9 (Fig. S3). In addition, we used medaka *leucophore free-2* (*lf-2*) as a *pax7a* mutant, which was previously reported as a loss-of-function mutation (Fig. S1) (Kimura et al., 2014).

**Fig. 2.**
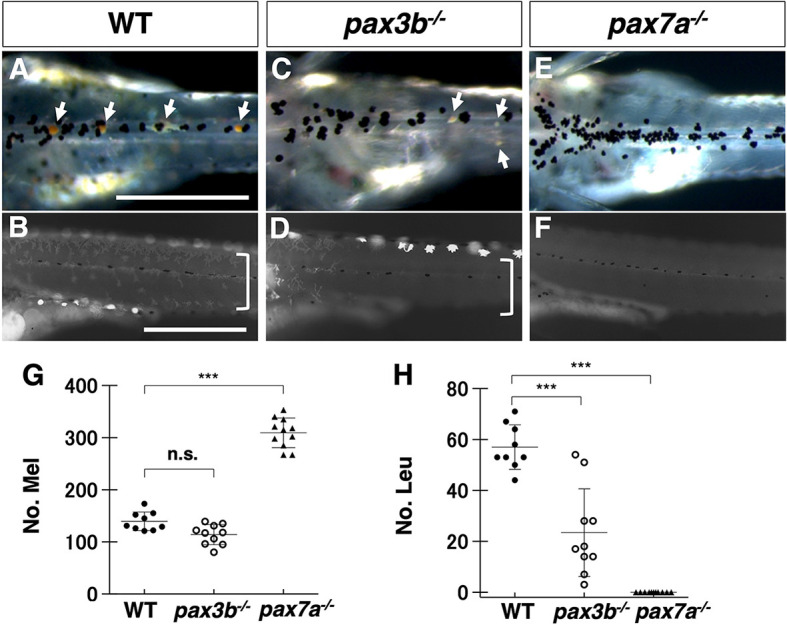
**Phenotypes of medaka *pax3* and *pax7* mutants.** (A-F) Medaka 9 dpf hatchlings. Comparison of pigment cell phenotypes between WT (A,B), *pax3b*^−/−^ (C,D) and *pax7a^−/−^* (*lf-2*) (E,F). (A,C,E) Dorsal views of the trunk in dark field. (B,D,F) Lateral views of the trunk under UV light. Images are representative of more than ten embryos. Scale bars: 250 µm. (G,H) Quantification of the number of melanophores (G) and leucophores (H) on the dorsal surface of the trunk. Note that leucophores are not always white but often yellow or orange, and have strong fluorescence under UV light. See ‘Microscopy’ in the Materials and Methods section. The highly fluorescent cells in the ventral edge in B and in the dorsal edge in D are leucophores. Xanthophores and leucophores were severely reduced in number in medaka *pax3b*^−/−^ mutant hatchlings (leucophores indicated by arrows in A and C; xanthophores, which are autofluorescent and dendritic under UV light, indicated by square brackets in B and D), and were completely absent in medaka *pax7a^−/−^* mutants (E,F,H). The number of melanophores was unaltered in *pax3b*^−/−^ mutants (A,C,G), but were significantly increased in *pax7a^−/−^* mutants (E,G). Significant difference was determined by Kruskal–Wallis test. ***P<0.05. *n*=8 for WT, 10 for *pax3b*, and 11 for *pax7a* in G,H. n.s., not significant. Error bars represent s.d.

We examined pigment cell phenotypes of the mutants. The medaka *pax3b* homozygous mutants showed a delay in the formation of xanthophores and leucophores (Fig. 2A-D, Fig. S4), which was detected as a reduction in the number of these cell types in hatchlings (Fig. 2G,H). This phenotype was much milder than that of the *pax7a* mutant, in which these two cell types were completely lost (Fig. 2E,F, Fig. S4) (Kimura et al., 2014). Medaka *pax3b* mutant fish became apparently normal in pigmentation as they grew, with xanthophores and leucophores restored (Fig. S4).

Similarly, zebrafish *pax3a* homozygous mutant hatchlings (Fig. S5) showed a delay in the formation of xanthophores, whereas xanthophores were completely and persistently lost in the *pax7a; pax7b* double mutant (Figs S5, S6). Melanophore number was not significantly altered in the absence of Pax3, but was increased in the absence of Pax7 in both medaka and zebrafish (Fig. 2G, Fig. S5J). The iridophore phenotype was faint and ambiguous in both medaka and zebrafish (Figs S4B,D,F, S5C,F,I,K).

The mutation in the paralogous gene, *pax3a* in medaka and *pax3b* in zebrafish, did not affect pigment cell development, or even enhance the phenotypes owing to the single paralogous mutation (medaka *pax3b^−/−^* and zebrafish *pax3a^−/−^*) (Figs S7 and S8).

In summary, the phenotypes due to loss of Pax3, by *pax3b* in medaka and *pax3a* in zebrafish, are most evident in xanthophores/leucophores and not significant or less severe in other cell types; notably, they do not include obvious melanophore phenotypes. Our results suggest that Pax3 is involved in the specification of xanthophores and leucophores in medaka, and xanthophores in zebrafish (which lack leucophores).

### Loss of Pax3 affects the expression of early xanthophore/leucophore markers

To investigate the early phenotypes of pigment cells in the absence of Pax3, we examined the expression pattern of specification markers. We used *GTP cyclohydrolase 2* (*gch2*) as a marker for xanthophore and leucophore progenitors (Pelletier et al., 2001), *dct* as a marker for melanophore progenitors (Kelsh et al., 2000) and *purine nucleoside phosphorylase* (*pnp4a*) as a marker for iridophore progenitors (Kimura et al., 2017; Petratou et al., 2021, 2018).

**Fig. 3.**
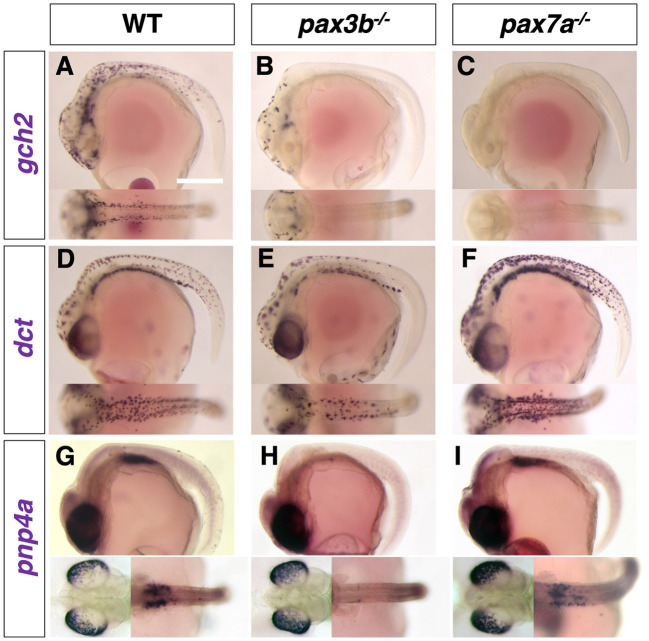
**Pigment cell progenitors in medaka *pax3b* and *pax7a* mutant embryos.** (A-I) Lateral views at top and dorsal views at bottom at stage 29. The *gch2*-expressing progenitors of xanthophore and leucophore were severely decreased in the *pax3b* mutant (A,B), with only a few remaining anteriorly, and completely absent in the *pax7a* mutant (C), which is consistent with the severity of the phenotypes in these mutants at later (hatching) stages. The *dct-*expressing progenitors of melanophore were slightly decreased in number in *pax3b* mutant (D,E), but unaltered or even increased in number and expression level in *pax7a* mutant compared with WT (F). The *pnp4a*-expressing progenitors of iridophore were not altered in the eyes in both mutants (G-I, bottom left), but the putative progenitors of peritoneum iridophores were decreased in the *pax3b* mutant (G,H), but not in the *pax7a* mutant (I, bottom right). Images are representative of more than ten embryos. Scale bar: 250 µm.

In medaka, expression of *gch2* in xanthophore/leucophore progenitors was severely reduced in the *pax3b* mutant (Fig. 3A,B) and completely absent in the *pax7a* mutant (Fig. 3C). Melanophore progenitors expressing *dct* appeared to be slightly reduced in number in the *pax3b* mutant (Fig. 3E), whereas they were unaltered or even increased in number and expression level compared with wild type (WT) in the *pax7a* mutant (Fig. 3D,F). Iridophore progenitors expressing *pnp4a* appeared to be reduced in the yolk sac, but not in the eyes in the *pax3b* mutant (Fig. 3G,H), whereas they are unaltered in the *pax7a* mutant (Fig. 3I). We obtained roughly similar results from zebrafish *pax3a* and *pax7a; pax7b* mutants (Fig. S9), except that *dct*-expressing melanophore progenitors were little changed by the loss of Pax3 (Fig. S9D,E) and *pnp4a*-expressing iridophore progenitors appeared to be slightly increased in the *pax7a; pax7b* mutant (Fig. S9G-I).

Like the phenotypes of pigmented cells in hatchlings, those of early specification markers in embryos suggest that the defects due to loss of Pax3 (Pax3a in zebrafish and Pax3b in medaka) are most manifest in the formation of xanthophores/leucophores.

### *pax3* functions upstream of *pax7*

**Fig. 4.**
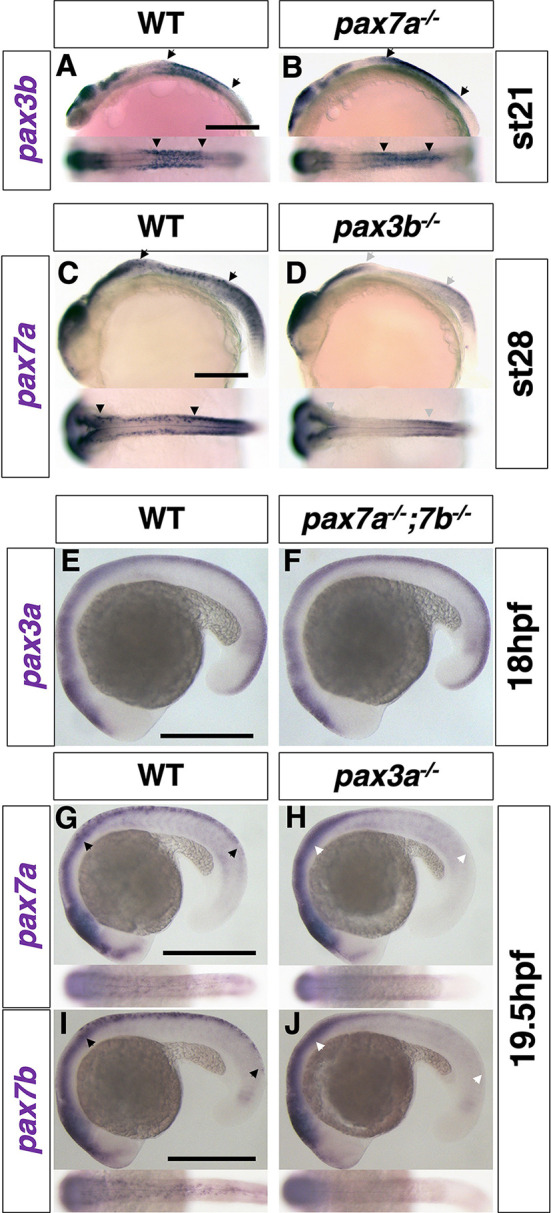
***pax3* functions upstream of *pax7*.** (A-D) Medaka. (A,B) *pax3b* expression at stage 21 (6 somite). (C,D) *pax7a* expression at stage 28 (30 somite). (E-J) Zebrafish. (E,F) *pax3a* expression at 18 hpf (18 somite). (G,H) *pax7a* expression at 19.5 hpf (21 somite). (I,J) *pax7b* expression at 19.5 hpf (21 somite). In medaka, *pax3b* expression was similar in WT and *pax7a* mutant embryos (A,B), whereas *pax7a* expression was largely reduced in NCCs in *pax3b* mutant embryos compared with WT (C,D). Similarly, in zebrafish, *pax3a* expression was not altered in *pax7a; pax7b* double-mutant embryos (E,F), but *pax7a* and *pax7b* expression was largely reduced in NCCs in *pax3a* mutant embryos compared with WT (G-J). Lateral views at top and dorsal views at bottom. Arrowheads indicate the anterior and posterior ends of the expression in the neural crest. Grey and white arrowheads indicate the absence of expression. Images are representative of more than ten embryos. Scale bars: 250 µm.

To investigate the genetic relationship between *pax3b* and *pax7a*, we examined the expression patterns of both *pax3b* and *pax7a* in the *pax7a* and *pax3b* mutants. *In situ* hybridization analyses showed that, whereas *pax3b*-expressing cells were unaffected in the *pax7a* mutant medaka (Fig. 4A,B), *pax7a*-expressing NCCs were severely reduced from the trunk region in *pax3b* mutant medaka embryos (Fig. 4C,D). Similarly, in zebrafish, both *pax7a* and *pax7b* expression was severely reduced in the *pax3a* mutant (Fig. 4E-J). These results suggest that, consistent with their expression timings, in the NCCs *pax3b* functions upstream of *pax7a* in medaka, that *pax3a* functions upstream of *pax7a* and *pax7b* in zebrafish, and also that *pax7* expression is partially dependent on *pax3*.

### The expression of *mitf* is dependent on *pax3*

**Fig. 5.**
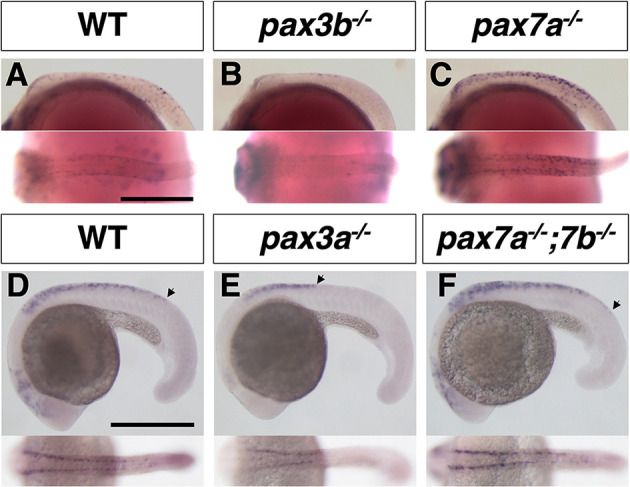
**Loss of *pax3* leads to a decrease in *mitfa* expression.** (A-C) Medaka embryos at stage 28 (30 somite). (D-F) Zebrafish embryos at 19.5 hpf (21 somite). Arrowheads indicate the posterior ends of *mitfa* expression in the neural crest (D-F). Lateral views at top and dorsal views at bottom. The *mitfa*-expressing cells were largely lost in the absence of Pax3 in medaka (A,B) and in zebrafish (D,E). Those cells appeared to be normal or rather increased in the medaka *pax7a* mutant (C) and in the zebrafish *pax7a; pax7b* double mutant (F). Images are representative of more than ten embryos. Scale bars: 250 µm.

The *dct* expression patterns in zebrafish and medaka Pax3 mutants are intriguing, because they suggest that the core role for Pax3 in melanocyte development in mammals has been conserved in medaka, but perhaps not in zebrafish, melanophores. To test directly the idea that the core GRN consisting of the transcription factors Sox10, Pax3 and Mitf plays a central role in melanophore development in teleosts, we assessed whether *mitf* expression might be dependent on Pax3 in medaka and zebrafish, similar to its role in mammals (Lang et al., 2005), by examining *mitfa* expression in medaka *pax3b* and zebrafish *pax3a* mutant embryos (Fig. 5). In comparison with medaka WT embryos (Fig. 5A), the *mitfa*-expressing cells were severely reduced in *pax3b* mutants (Fig. 5B), whereas they were comparable or rather increased in *pax7a* mutant embryos (Fig. 5C). Similarly, in zebrafish at 19.5 hpf, early *mitfa* expression was partially lost in *pax3a* mutant (Fig. 5D,E), but not in *pax7a; pax7b* double-mutant embryos (Fig. 5F). The expression pattern in zebrafish in particular indicates that the Pax3 mutation might cause delayed upregulation of *mitfa*, whereas the loss of Pax7 activity might allow precocious upregulation of *mitfa*. Overall, and importantly, these results indicate that loss of Pax3 causes a reduction in *mitfa* expression in medaka and zebrafish, suggesting that *mitfa* transcription is partially dependent on Pax3 activity.

### Cooperative function of Pax3 and Sox10 can promote *mitf* expression

**Fig. 6.**
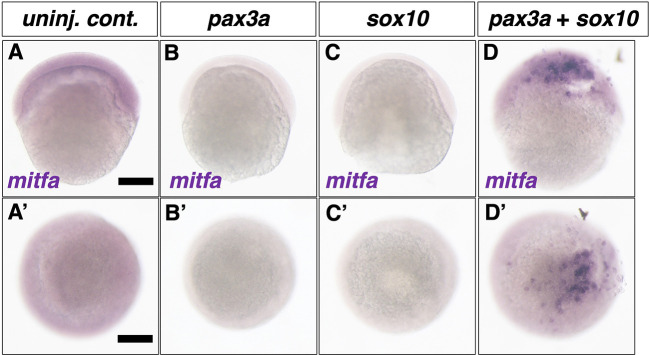
**Overexpression of *pax3a* and *sox10* induces ectopic expression of *mitfa* in zebrafish.** (A-D′) *mitfa* expression at 6 hpf in control (A,A′), *pax3a* synthetic RNA-injected (B,B′), *sox10* synthetic RNA injected (C,C′) and *pax3a* and *sox10* synthetic RNA co-injected (D,D′) embryos. (A-D) Lateral views. (A′-D′) Animal pole views. *mitfa* mRNA was not expressed endogenously in 6 hpf embryo (A). Whereas overexpression of *pax3a* or *sox10* by synthetic RNA injection into 1- to 2-cell-stage embryos failed to induce *mitfa* expression at 6 hpf, simultaneous overexpression of *pax3a* and *sox10* can induce ectopic expression of *mitfa*. Scale bars: 200 µm. Fractions were 23/23 for *pax3a* injection, 16/16 for *sox10* injection and 30/38 for *pax3a* and *sox10* co-injection.

To investigate whether Pax3 directly controls *mitf* transcription in teleosts, we performed a Pax3 overexpression experiment by synthetic RNA injection into zebrafish embryos. Working on the assumption that Pax3 cooperates with Sox10 to activate *mitf* transcription, as in mice, we injected synthetic RNAs of *pax3a* and/or *sox10* into one-cell-stage embryos and examined whether *mitfa* mRNA was ectopically expressed in the injected embryos at 6 h post-fertilization (hpf), when *mitfa* mRNA is not yet endogenously expressed in zebrafish (Fig. 6A,A′).

Whereas *mitfa* mRNA was not detected *in situ* when Pax3a (Fig. 6B,B′) or Sox10 (Fig. 6C,C′) were overexpressed alone, simultaneous overexpression of these transcription factors ectopically induced *mitfa* expression at a high rate (Fig. 6D,D′). Our results suggest that the cooperative action of Pax3 and Sox10 to promote *mitf/Mitf* expression is conserved between zebrafish and mice (Lang et al., 2005).

### Mitfa can drive the progenitor markers for melanophore and xanthophore

**Fig. 7.**
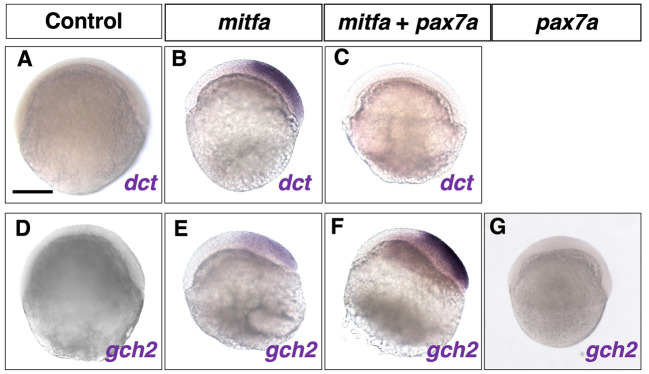
**Overexpression of *mitfa* induces ectopic expression of progenitor markers for melanophore and xanthophore.** (A-G) *dct* (A-C) and *gch2* (D-G) expression in 6 hpf control (A,D), *mitfa* synthetic RNA-injected (B,E), *mitfa* and *pax7a* synthetic RNA-injected (C,F) and *pax7a* synthetic RNA-injected (G) embryos. Overexpression of *mitfa* alone can strongly induce ectopic *dct* expression (A,B; 22/23). Simultaneous overexpression of *pax7a* suppresses the ectopic *dct* expression by *mitfa* (C; 18/18). Ectopic expression of *gch2* mRNA is induced by overexpression of *mitfa* alone (D,E; 20/24) and more strongly induced by overexpression of *mitfa* and *pax7a* in combination (F; 26/31). Overexpression of *pax7a* alone is unable to induce ectopic *gch2* expression (G). Scale bar: 250 µm.

The results above suggest that the GRN consisting of Sox10, Pax3 and Mitf is conserved in zebrafish, where Mitfa plays an essential role in melanophore differentiation (Greenhill et al., 2011). However, loss of Pax3 has the most severe effect on xanthophore formation in zebrafish and medaka. Given that Mitf mediates Pax3 function, we hypothesized that Mitf is involved in regulating, not only melanophore differentiation, but also xanthophore differentiation in zebrafish. To test this, we examined whether *mitfa* overexpression could induce ectopic expression of markers for melanophore (*dct*) and xanthophore (*gch2*) progenitors in zebrafish embryos (Fig. 7). The *dct* and *gch2* mRNAs were not expressed endogenously in control 6 hpf embryos (Fig. 7A,D). Injection of *mitfa* synthetic RNA induced ectopic mRNA expression of *dct* (Fig. 7B), but also of *gch2* (Fig. 7E). Thus, it is likely that Mitfa can promote both xanthophore and melanophore fates in zebrafish when overexpressed.

### Pax7 can suppress Mitf action to drive *Gch2* transcription

What determines the fate choice of the *mitfa*-expressing progenitors to either melanophore or xanthophore fate? To address this, we focused on the previous report in mice that Pax3 can inhibit Mitf-mediated transcriptional activation of *Dct* after activating *Mitf* transcription in cooperation with Sox10 (Lang et al., 2005). We hypothesized that after Pax3 has activated *mitfa* expression, Pax7 instead of Pax3 might play the inhibitory role against Mitfa-driven transcription of *dct* in zebrafish. To test this hypothesis, we examined whether Pax7 could affect the ectopic expression of *dct* and *gch2*, when co-overexpressed with Mitfa in zebrafish embryos. The ectopic expression of *dct*, which was detected in the *mitfa*-injected embryos, was lost in the 6 hpf embryos co-injected with *mitfa* and *pax7a* RNAs (Fig. 7C). By contrast, *pax7a* co-injection enhanced the ectopic expression of *gch2* in the *mitfa*-injected 6 hpf embryos (Fig. 7F). Interestingly, injection of *pax7a* synthetic RNA alone was unable to activate ectopic *gch2* expression (Fig. 7G).

### Mitf specifies the fate of melanophore and xanthophore in medaka

To determine whether Mitf activity is required not only for melanophore fate but also for xanthophore fate, we attempted to generate loss-of-function mutants for Mitf in medaka. We found two *mitf* paralogous genes, *mitfa* and *mitfb*, in the medaka genome, as in zebrafish (Lister et al., 2001). The expression pattern of these genes, examined by *in situ* hybridization, showed that both appeared to be expressed in the NCCs (Fig. S10). We therefore induced mutations in *mitfa* and *mitfb* using CRISPR/Cas9, targeting the region encoding the bHLH domain (Fig. S11).

**Fig. 8.**
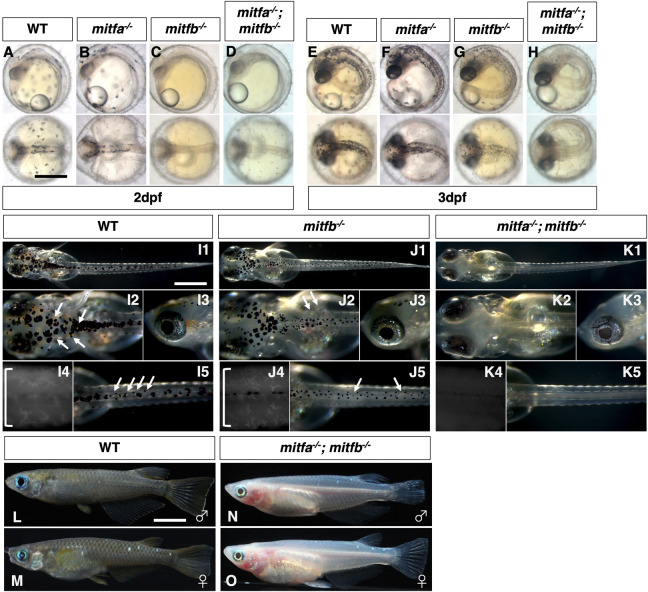
**Phenotypes of medaka *mitfa* and *mitfb* double homozygotes.** (A-O) Phenotypes of wild-type (WT), *mitfa* single, *mitfb* single and *mitfa* and *mitfb* double mutants in 2 dpf (A-D) and 3 dpf (E-H) embryos, 7 dpf hatchlings (I-K) and 4-month-old adult male (L,M) and female (N,O) fish. (I4,J4,K4) Autofluorescence images showing xanthophores under UV light. (A-H) Upper panels are dorsal views; lower panels are lateral views. (G-K) Panels 1, 2 and 5 are dorsal views; panels 3 and 4 are lateral views. (L-O) Lateral views. Melanophores first appear on the head, the anterior body and the yolk at 2 dpf (A) and increase in number to become distributed throughout the body at 3 dpf (E) in the WT embryo. The *mitfa* mutant embryo looks normal at this time (B,F), whereas the *mitfb* mutant does not have melanophores at 2 dpf (C), but shows their delayed formation at 3 dpf (G). The *mitfa; mitfb* double mutant completely lacks melanophores during this period and thereafter (D,H). At the hatching stage, all the four types of pigment cells are differentiated (pigmented) in WT (I1-I5). The *mitfb* mutant looks normal except that melanophores are relatively small and few leucophores are found (arrows in J2,J5) compared with those in WT (I1-I5). The *mitfa; mitfb* double mutant completely lacks not only melanophores (K1,K2,K5) but also xanthophores (K4) and leucophores (K1,K2,K5), but retains iridophores in the eyes and on the yolk (K1,K2,K3). Square brackets indicate xanthophores on the lateral surface of the body (I4,J4). In adulthood, compared with WT (L,M), it is obvious that the *mitfa; mitfb* double mutant lacks all visible pigmentation except for that of iridophores in the skin and the iris (N,O). Scale bars: 0.5 mm (in A for A-H, in I1 for I1-K1); 5 mm (in L for L-O).

We successfully generated mutations in the *mitfa* and *mitfb* genes in medaka (Fig. S11). Single loss of *mitfb* resulted in a defect in melanophore formation, being delayed at 2 days post-fertilization (dpf) (Fig. 8A,C) and partially restored at 3 dpf (Fig. 8E,G) and at hatching (Fig. 8I,J), whereas loss of *mitfa* did not (Fig. 8B,F), suggesting that *mitfb*, but not *mitfa*, plays the more central role in melanophore development in medaka. The double loss of *mitfa* and *mitfb* resulted in complete absence of not only melanophores but also xanthophores and leucophores throughout embryogenesis (Fig. 8D,H,K), and even later during adulthood (Fig. 8L-O). Iridophore formation appeared normal throughout life (Fig. 8K2,K3,N,O). These data suggest that Mitfa and Mitfb are redundant in the NCCs and, together, are essential for the development of melanophores, xanthophores and leucophores in medaka.

**Fig. 9.**
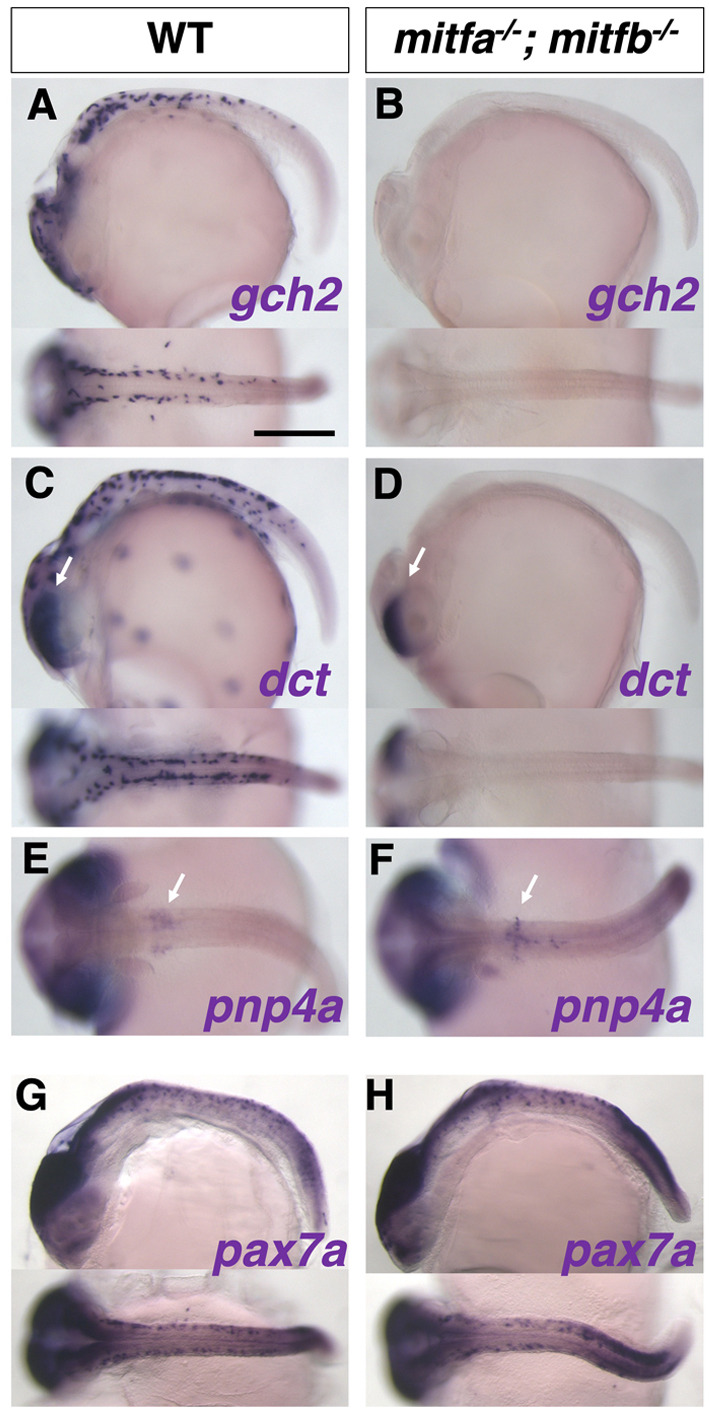
***In situ* analyses of medaka *mitfa^−/−^*; *mitfb^−/−^* double mutants with pigment cell markers.** Results of *in situ* analyses with specification markers, *dct* for melanophore, *gch2* for xanthophore/leucophore, and *pnp4a* for iridophore, are consistent with the phenotypes of differentiated pigment cells. The *gch2*-expressing xanthophore/leucophore progenitors are lost in the double mutant (A,B). The *dct*-expressing melanophore progenitors are absent from the body surface, but not from the eyes (retinal pigment epithelium, RPE) in the double-mutant embryo (C,D; arrows indicate the signal in RPE), consistent with the phenotype that the mutant retains melanized RPE (see Fig. 8K1,K3). The *pnp4a*-expressing iridophore progenitors appear unchanged in the double mutant compared with WT (E,F; arrows indicate the signal on the yolk). The *mitfa^−/−^*; *mitfb^−/−^* double mutant shows normal expression pattern of *pax7a* mRNA compared with WT (G,H), suggesting that the defect in xanthophore and leucophore formation in the double mutant is not mediated by Pax7a function. Lateral views at top and dorsal views at bottom. (A-D,G,H) Stage 28. (E,F) Stage 29. Scale bar: 250 µm.

*In situ* hybridization analyses support the idea that Mitfs are not only required for melanophore fate but also for specification of xanthophore/leucophore fate (Fig. 9). Expression of *gch2* and *dct* was absent from the body surface in the *mitfa*; *mitfb* double homozygotes (Fig. 9A-D). In contrast, *pnp4a* mRNA was expressed in the eyes and on the yolk similarly in WT and the *mitfa*; *mitfb* double mutant (Fig. 9E,F). These results are fully consistent with the above phenotype that the double mutant has only iridophores but lacks the other three types of pigment cells.

To exclude the possibility that Mitfs regulate *pax7a* expression to activate the transcription of *dct* and *gch2*, we examined the expression of *pax7a* mRNA in the *mitfa*; *mitfb* double homozygotes. The *pax7a*-expressing cells were not notably altered in the double mutant (Fig. 9H) compared with WT (Fig. 9G), suggesting that Mitfs directly activate the transcription of *dct* and *gch2*, and that, consistent with the overexpression results in Fig. 7, Pax7a alone is not sufficient to drive *gch2* transcription.

**Fig. 10.**
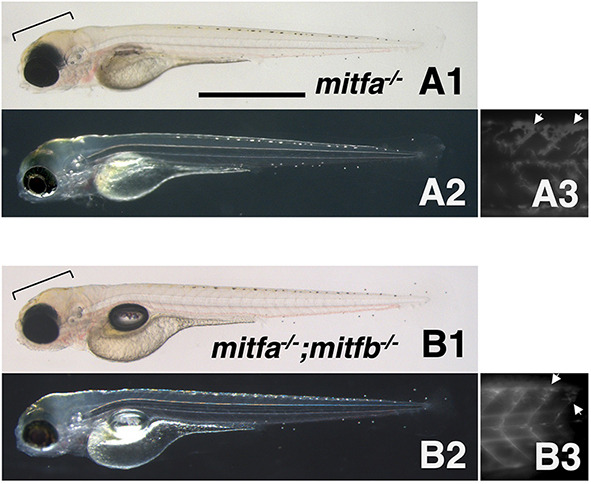
**Phenotypes of zebrafish *mitfa* and *mitfb* double homozygotes.** (A1-B3) 4 dpf *mitfa^−/−^* (*nacre*) hatchling (A1-A3) and 4 dpf *mitfa^−/−^*; *mitfb^−/−^* hatchling (B1-B3) under normal transmission optics (A1,B1), dark-field epi-illumination optics (A2,B2) and autofluorescence images showing xanthophores under UV light (A3,B3). Yellowish-pigmented xanthophores are observed in the dorsal head of the *mitfa^−/−^* hatchling (A1,A2). Autofluorescence emitted by xanthophores is clearly visible in the trunk of *mitfa^−/−^* (A3). Similarly, the *mitfa^−/−^*; *mitfb^−/−^* hatchling has xanthophores in the head (B1,B2) and autofluorescent cells in the trunk (B3). Brackets indicate pigmented xanthophores and arrows indicate autofluorescent (possibly immature) xanthophores. Scale bar: 1 mm.

Finally, to assess whether the function of Mitf is conserved among fish species, we generated zebrafish *mitfa^−/−^*; *mitfb^−/−^* double mutants by additionally disrupting the *mitfb* gene by CRISPR/Cas9 in the *mitfa^nacre^* background (Fig. S12) and observed the double-mutant phenotype (Fig. 10). Compared with a single *mitfa* mutant, *mitfa^−/−^*; *mitfb^−/−^* double mutants showed a slightly more severe reduction of xanthophores, with fewer fluorescent xanthophores on the trunk (Fig. 10A3,B3). However, unlike the medaka *mitfa*; *mitfb* double homozygotes, the zebrafish double mutants retained xanthophores on the head (Fig. 10A1,B1). Thus, the results suggest that in zebrafish xanthophore development may be partially dependent on Mitf function, but that some other factor(s) may compensate for the loss of Mitfs.

## DISCUSSION

We established medaka and zebrafish mutants of transcription factors that we expected to be involved in the GRN of pigment cell-fate specification. Their phenotypes are summarized in Table 1.

**Table 1.**
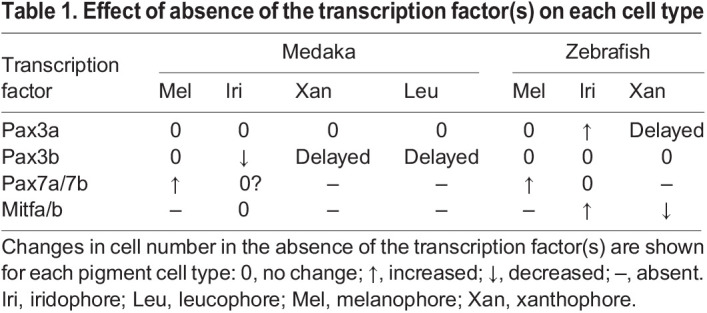
Effect of absence of the transcription factor(s) on each cell type

Although Pax7 has been considered a requisite for xanthophore differentiation in medaka and zebrafish and additionally for leucophore differentiation in medaka (Kimura et al., 2014; Minchin and Hughes, 2008; Nord et al., 2016), Pax3, paralogous to Pax7, has not yet been extensively studied in the context of pigment cell development. In this study, we elucidated the function of Pax3 in pigment cell development and proposed that the set of transcription factors Sox10, Pax3 and Mitf regulate the melanophore and xanthophore fates (plus leucophore fate in medaka). Furthermore, we found that the choice of melanophore versus xanthophore/leucophore fate appears to be regulated by the interaction of Mitf and Pax7. In the absence of Pax7, Mitf strongly drives the melanophore progenitor gene *dct*, whereas Mitf and Pax7 cooperatively drive the xanthophore/leucophore progenitor gene *gch2*.

### Partially overlapping but sequential function of Pax3 and Pax7

Pax3 is expressed and functions before Pax7 during NCC development, and appears to be temporally restricted to the initial phase of pigment cell progenitor formation. Pax7 expression occurs slightly later than, but overlaps with, Pax3 expression, and is long maintained until a later stage of cell differentiation (Fig. 1, Fig. S2). Assuming that Pax3 and Pax7 are highly homologous and have similar activities (Pax3a versus Pax7a/7b= 96.8% similarity; Pax7a versus Pax7b=100% in homeodomain of zebrafish proteins), loss of Pax3 might be partially compensated by Pax7 at early stages, but loss of Pax7 cannot be compensated by Pax3 at later stages because Pax3 is no longer expressed. The mutant phenotypes support this idea: xanthophores and leucophores are partially formed in the absence of Pax3, whereas these pigment cell types are completely lost in the absence of Pax7, in medaka (Fig. 2, Fig. S4). This is also the case for xanthophores in zebrafish (Figs S5, S6).

### The Sox10, Pax3 and Mitf GRN in teleosts

Pax3 is required for activation of *mitf* expression, as shown in the medaka *pax3b* mutant and zebrafish *pax3a* mutant with delayed *mitfa* expression (Fig. 5). Overexpression of Pax3 together with Sox10 resulted in ectopic expression of *mitfa* in zebrafish embryos (Fig. 6). As a previous report using human cultured cells showed that Pax3 functions with Sox10 to activate *Mitf* expression (Lang et al., 2005), we propose that a conserved role of Pax3 in vertebrates is to form a core melanophore GRN with Sox10 to regulate Mitf in the NCCs.

The medaka and zebrafish *pax3* mutants only showed ambiguously the melanophore-related phenotypes. Although loss of Pax3 appeared to substantially decrease *mitf* expression, it eventually resulted in only partially or barely reduced melanophore formation in both medaka and zebrafish (Fig. 2, Figs S4-S6). We reason this can be explained as *mitf* expression is delayed in the absence of Pax3 and perhaps subsequently compensated by some other transcription factors. In contrast, loss of Pax7 resulted in significant increase of *mitfa*-expression in progenitors and pigmented melanophores in both species (Fig. 2, Figs S4-S6). As Pax7, and presumably Pax3, inhibit Mitf protein from promoting melanophore fate (see below), loss of Pax3 or Pax7 would tend to increase melanophore formation (Fig. 2, Fig. S5). These conflicting actions may explain the ambiguous melanophore phenotypes in *pax3* mutants.

### Pax7 interacts with Mitf to segregate pigment cell lineages

In mice, Mitf directly activates *Dct* expression and drives the cells to differentiate into melanocytes (Lang et al., 2005; Ludwig et al., 2004). In teleosts, Mitf also drives *dct* expression, reflecting a broader role in driving melanophore differentiation (Elworthy et al., 2003; Greenhill et al., 2011; Lister et al., 1999). However, here we show that co-expression of Pax7 can inhibit Mitf from driving *dct* transcription, which we suggest has physiological relevance when *pax7* is expressed in a subset of the *mitfa*-expressing NCC population (Fig. 7). In these cells, *gch2* is activated by the cooperative action of Pax7 and Mitfa instead of *dct* (Fig. 7), presumably reflecting a broader role for the combinatorial impact of these transcription factors, which together drive differentiation into xanthophores or leucophores. If *pax7* is not co-expressed, the *mitfa*-expressing cells would differentiate into melanophores, as in mice. We still do not know how the *mitfa*-expressing progenitor population is divided into two, one expressing Pax7 and the other not.

### Model

**Fig. 11.**
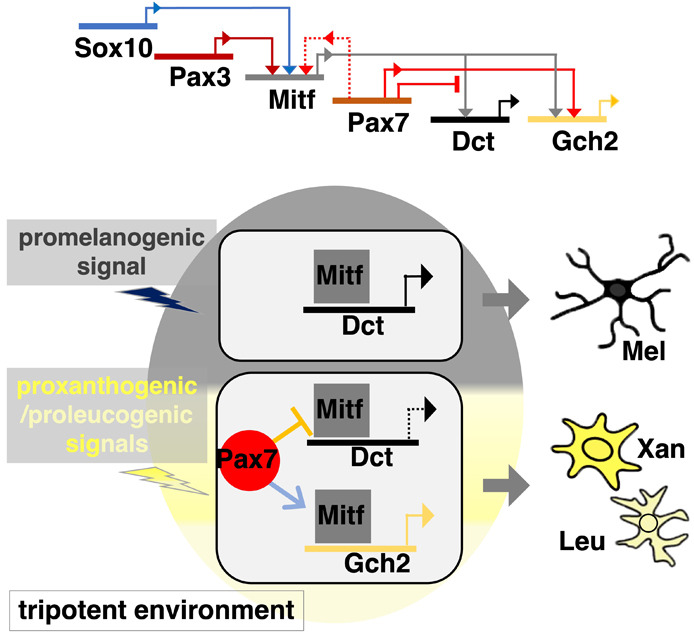
**Model of a gene regulatory network.** Our results lead us to postulate a GRN that controls the development of melanophores with Dct expression and xanthophores/leucophores with Gch2 expression. In medaka NCCs, Sox10 (Sox10a and Sox10b in medaka) and Pax3 (Pax3b in medaka) activate Mitf (Mitfa and possibly Mitfb) expression. Mitf can drive transcription of Dct and Gch2. Pax7 represses transcription of Dct by inhibiting Mitf, and promotes transcription of Gch2 cooperatively with Mitf. Pax7 may compensate for loss of Pax3 by activating Mitf expression (dotted line). Expression of Mitf and Pax7 are dependent upon promelanogenic and proxanthogenic/proleucogenic signals, respectively (the cell resides in a tripotent environment). A fraction of the Mitf-expressing cells, if expressing Pax7a (lower cell), would differentiate into the Gch2-expressing xanthophore/leucophore lineage because Mitf and Pax7 cooperatively activate Gch2 expression, whereas Pax7 represses Dct by inhibiting Mitf. Another cell, not expressing Pax7 (upper cell), would differentiate into the Dct-expressing melanophore lineage where Mitf activates Dct expression. Leu, leucophore; Mel, melanophore; Xan, xanthophore.

We propose that a more complex GRN than in mammals controls the fate specification of melanophore progenitors and xanthophore/leucophore progenitors in medaka (Fig. 11). Pax3 and Sox10 activate *mitf* expression in the NCC-derived multipotent progenitors for pigment cells. The fate of the *mitf*-expressing progenitor cells is determined by whether or not they express Pax7. These progenitors, if expressing Pax7, give rise to xanthophores and leucophores in medaka (to xanthophores in zebrafish), or, if not expressing Pax7, they give rise to melanophores.

Previously, we and others have explained pigment cell development from NCCs in terms of a progressive fate restriction model, postulating partially restricted intermediates, such as a bipotent progenitor of melanophores and iridophores and a bipotent progenitor of xanthophores and leucophores. Recently, based upon detailed evaluation of pigment cell development in the zebrafish, we have proposed an alternative view, the cyclical fate restriction (CFR) model. Under CFR, we predict that pigment cell development proceeds directly and dynamically from a highly multipotent state (Kelsh et al., 2021; Subkhankulova et al., 2023). We consider that this view can be readily applied to the situation in medaka too. The key is to replace the concept of a bipotent or tripotent progenitor with that of a bipotent or tripotent environment – one in which the broadly multipotent cell is exposed to two or three key fate-determining signals, which limit the options available to the multipotent cell in that region. In this context, we suggest that a pigment cell progenitor expresses *mitf* in response to a promelanogic environment. But, in addition, if the cell receives unknown signals that are xanthogenic and leucogenic (a tripotent environment), and when these are strong or prolonged enough, *pax7* expression becomes dominant, and the cell adopts a xanthophore or leucophore fate. If the signals are too transient/weak, the cell would differentiate into a melanophore.

To our knowledge, Pax7 has not been implicated in pigment cell development in mammals and birds, which lack xanthophores, whereas Pax3 plays a key role in melanocyte development in mammals (Conway et al., 1997; Tassabehji et al., 1992). It is conceivable that the sequential activity of earlier Pax3 and later Pax7 is key to the development of xanthophores and leucophores in vertebrates, and also that loss of Pax3-dependent Pax7 expression may contribute to the evolutionary loss of xanthophore fate from the endothermic vertebrates.

### Mitf is a key player in the formation of both melanophore and xanthophore progenitors

Overexpression of Mitfa alone can ectopically activate the expression of not only *dct* but also *gch2* in zebrafish embryos. This suggests an unexpected role of Mitfa in promoting the xanthophore fate. Indeed, our genetic study in medaka showed that Mitfs (Mitfa and Mitfb) are essential for the formation of, not only melanophores, but also xanthophores and leucophores (Figs 8, 9).

Mitf has long been believed to be the master regulator of melanophores in teleosts as in mammals (Arnheiter, 2010) because zebrafish *mitfa* mutants lack melanophores completely (Lister et al., 1999). At the same time, however, xanthophore pigmentation becomes reduced in the *mitfa* mutant, implying that Mitf is involved in xanthophore formation and that, perhaps, Mitfb compensates for the loss of Mitfa. Lister et al. generated *mitfa; mitfb* double mutants, but did not describe whether they lacked xanthophores (Lister et al., 2001).

Our study clearly shows that loss of Mitfa and Mitfb results in complete absence of melanophores, xanthophores and leucophores throughout life in medaka (Fig. 8), suggesting that Mitfs (Mitfa and Mitfb) are required for fate specification of, not only melanophores, but also xanthophores and leucophores in medaka. In contrast, zebrafish *mitfa*; *mitfb* double mutants retain xanthophores, as we confirmed by using the *mitfb* allele we generated in combination with the *mitfa^nacre^* allele (Fig. S12, Fig. 10). It remains unclear why Mitf loss-of-function phenotypes differ between zebrafish and medaka with respect to xanthophore formation. However, the slightly enhanced reduction of xanthophores by loss of Mitfb in the zebrafish *mitfa/nacre* mutant suggests that Mitf activity is also involved in xanthophore development in zebrafish (Fig. 10). We speculate that Tfec, a transcription factor homologous to Mitf, which is required for iridophore specification, might partially compensate for the loss of Mitfa and Mitfb to form xanthophores on the head in zebrafish. Indeed, Tfec and Mitfs are partially redundant for pigment cell development in zebrafish (Petratou et al., 2021; Subkhankulova et al., 2023). We speculate that the degree of redundancy of Mitf and Tfec may differ between medaka and zebrafish, so that the formation of xanthophores in these species shows differential regulation by Mitf and Tfec.

In conclusion, the core GRN consisting of Sox10, Pax3 and Mitf to regulate pigment cell development is conserved across teleosts and mammals. We propose that the core GRN governs melanophore and xanthophore/leucophore potentials in teleosts. Recruitment of Pax7 to the GRN allows teleosts to create cell diversity, e.g. xanthophores/leucophores, implying an evolutionary aspect of cell diversification in vertebrates. Finally, although Mitf is considered to be a master regulator of the melanocyte phenotype, this picture may be rather misleading outside of a mammalian context; instead, we note that our results and previous studies make it clear that pigment cell fate determination in fish likely reflects the combinatorial activity of multiple transcription factors, including those of the Mitf/Tfec, Pax and Sox families. Further work will be required to define fully the transcription factor complements responsible for each cell type.

## MATERIALS AND METHODS

### Ethics

The animal work in this study was approved by the Nagoya University Animal Experiment Committee and was conducted in accordance with the Regulations on Animal Experiments at Nagoya University.

### Strains and fish husbandry

The Nagoya and d-rR strains of the medaka fish *Oryzias latipes* were used as the WT (Nagao et al., 2014; Yamamoto, 1953). Medaka *pax3a* and *pax3b* mutant strains were generated by TALENs in the d-rR strain and maintained in the d-rR background, designated as d-rR; *pax3a^ex2del5^* and d-rR; *pax3b^ex2del11^*. The d-rR; *pax3b^ex2del11^* strain was crossed with the Nagoya strain to obtain Nagoya; *pax3b^ex2del11^* having melanized melanophores (see Figs S4, S7). The *pax3a^ex2del5/ex2del5^* and *pax3b^ex2del11/ex2del11^* mutants are considered null mutants and are therefore referred to as *pax3a^−/−^* and *pax3b^−/−^*, respectively. The medaka *leucophore free-2* strain, previously described (Kimura et al., 2014), was used as *pax7a* null mutant. The medaka *mitfa^ex6del1^* and *mitfb^ex6del7^* mutation was generated by CRISPR/Cas9 on the Nagoya background. In this work, the homozygous mutants are designated as *mitfa^−/−^* and *mitfb^−/−^*.

The AB strain of the zebrafish *Danio rerio* was used as the WT. Zebrafish *pax3a*, *pax3b* and *pax7a* mutant strains were generated by TALENs and *pax7b* mutant by CRISPR/Cas9. The alleles isolated in this study are *pax3a^ex2del14^*, *pax3b^ex2del11^*, *pax3b ^ex2del16^*, *pax7a^ex2del19^* and *pax7b^ex1del10^*. All the mutant alleles are considered null alleles, and thus these zebrafish mutants are designated as *pax3a^−/−^*, *pax3b^−/−^*, *pax7a^−/−^* and *pax7b^−/−^*.

### Genotyping

Mutations in medaka and zebrafish were detected using PCR fragment length polymorphism by polyacrylamide gel electrophoresis (PAGE), as previously described (Nagao et al., 2018). PCR primer sets were: medaka *pax3a^ex2del5^*, 5′-AGGTCTCTGGATTTTTCTAACCTAAACCCG-3′ and 5′-TGGTGCGCCATCTCCACGATCTTATG-3′; medaka *pax3b^ex2del11^*, 5′-TGCTGTGATTGAACGCAGTGTCCACCCCGC-3′ and 5′-GTCTCCTGGTACCGGCACAGGATTTTGGAC-3′; zebrafish *pax3a^ex2del14^*, 5′-CGCTGACTTTTCCTCTTTTGT-3′ and 5′-GCGGATGTGATTGGGCAAAGG-3′; zebrafish *pax3b^ex2del11 or ex2del16^*, 5′-TGTCAACTCCGATGGGTCAG-3′ and 5′-AGAGACTCTGAGCTGGCGGG-3′; zebrafish *pax7a^ex2del19^*, 5′-TCTCAACACCTCTGGGTCAAG-3′ and 5′-CCATTTCCACTATTTTGTGTC-3′; zebrafish *pax7b^ex1del10^*, 5′-GTAGAATGTCATCCTTACCGG-3′ and 5′-CTTCCAAGGGGAATCCGGTGC-3′; medaka *mitfa^ex6del1^* 5′-AGGTTCAACATTAATGACCGCATT-3′ (WT allele-specific sense) and 5′-AGGTTCAACATTAATGACCGCATA-3′ (mutant allele-specific sense) and 5′-TGATTGCAGATGATTTGCCCATC-3′ (common antisense); medaka *mitfb^ex6del7^*, 5′-GCATTGAAGCATTTCTTCCAG-3′ and 5′-AAAATGGCGGGATATACGTAC-3′. PCR products were separated by PAGE to detect the deletion in size or by agarose gel to check for presence or absence (for *mitfa^ex6del1^*).

### TALEN- and CRISPR/Cas9-mediated mutagenesis

Mutagenesis was carried out according to methods described previously, e.g. for TALEN (Ansai et al., 2013) and for CRISPR/Cas9 (Ansai and Kinoshita, 2014). The internal spacer sequences of a pair of TALEN targets were: medaka *pax3a*, 5′-CAACCAGCTCGGCGG-3′; medaka *pax3b*, 5′-TTGCCTAACCATATCCG-3′; zebrafish *pax3a*, 5′-GGGCCGCGTGAACCA-3′; zebrafish *pax3b*, 5′-TCTGCCCAACCACATTC-3′; zebrafish *pax7a*, 5′-TTTTCATCAACGGA-3′.

The target sequences of CRISPR/Cas9 were: zebrafish *pax3b*, 5′-ATCACGGAATCCGGCCCT-3′; zebrafish *pax7b*, 5′-TACCGCGAATGATGCGAC-3′. We purchased crRNA for targeting medaka *mitfa* and *mitfb*: *mitfa*, 5′-AGGTTCCTAGTTCCTTAATGCGG-3′; *mitfb*, 5′-GAAGGTTCAACATCAACGATCGG-3′ (Integrated DNA Technologies), and injected each with tracrRNA and Cas9 protein (Integrated DNA Technologies) into one-cell embryos.

### Transcript detection in whole-mount embryos

Whole-mount *in situ* hybridization was performed as previously described (Nagao et al., 2014). The digoxigenin (Roche Diagnostics GmbH)-labelled antisense riboprobe was synthesized from a plasmid containing the full- or partial-length open reading frame of the cDNAs using SP6 or T7 polymerase (Promega) after restriction enzyme digestion (New England Bio Labs).

### Microscopy

Melanophores were examined under a stereomicroscope (MZ APO, Leica Microsystems) using a combination of bright- and darkfield illumination. Leucophores and iridophores were identified in dark field. Xanthophores were identified by detecting their autofluorescence under UV light with a DAPI filter (Imager.D1, Carl Zeiss).

Note that larval leucophores appear orange to varying degrees and are clearly visible under darkfield illumination, whereas xanthophores appear yellowish but are ambiguous under brightfield illumination. Under UV light, leucophores show stronger autofluorescence than xanthophores, but iridophores and melanophores do not. Leucophores and xanthophores can be distinguished by their location: leucophores are located along the dorsal and ventral midlines, whereas xanthophores are located laterally across the body surface in the larva.

A Zeiss LSM700 confocal microscope was used to observe GFP fluorescent cells in the transgenic embryos.

### Constructs and microinjection of synthetic RNAs

The full-length open reading frame of the cDNA for each gene was subcloned into a pCS2 plasmid (Turner and Weintraub, 1994). Artificial mRNA (synthetic RNA) was transcribed *in vitro* using SP6 polymerase (Promega) after NotI digestion (New England Bio Labs).

## Supplementary Material

10.1242/develop.202114_sup1 Supplementary information
