# Supplementary material for: A gene regulatory network combining Pax3/7, Sox10 and Mitf generates diverse pigment cell types in medaka and zebrafish
Source: Development. 2023 Oct 12;150(19):dev202114. doi: 10.1242/dev.202114 (PMC10617610; doi:10.1242/dev.202114)
Supplement: Supplementary information [file develop-150-202114-s1.pdf]

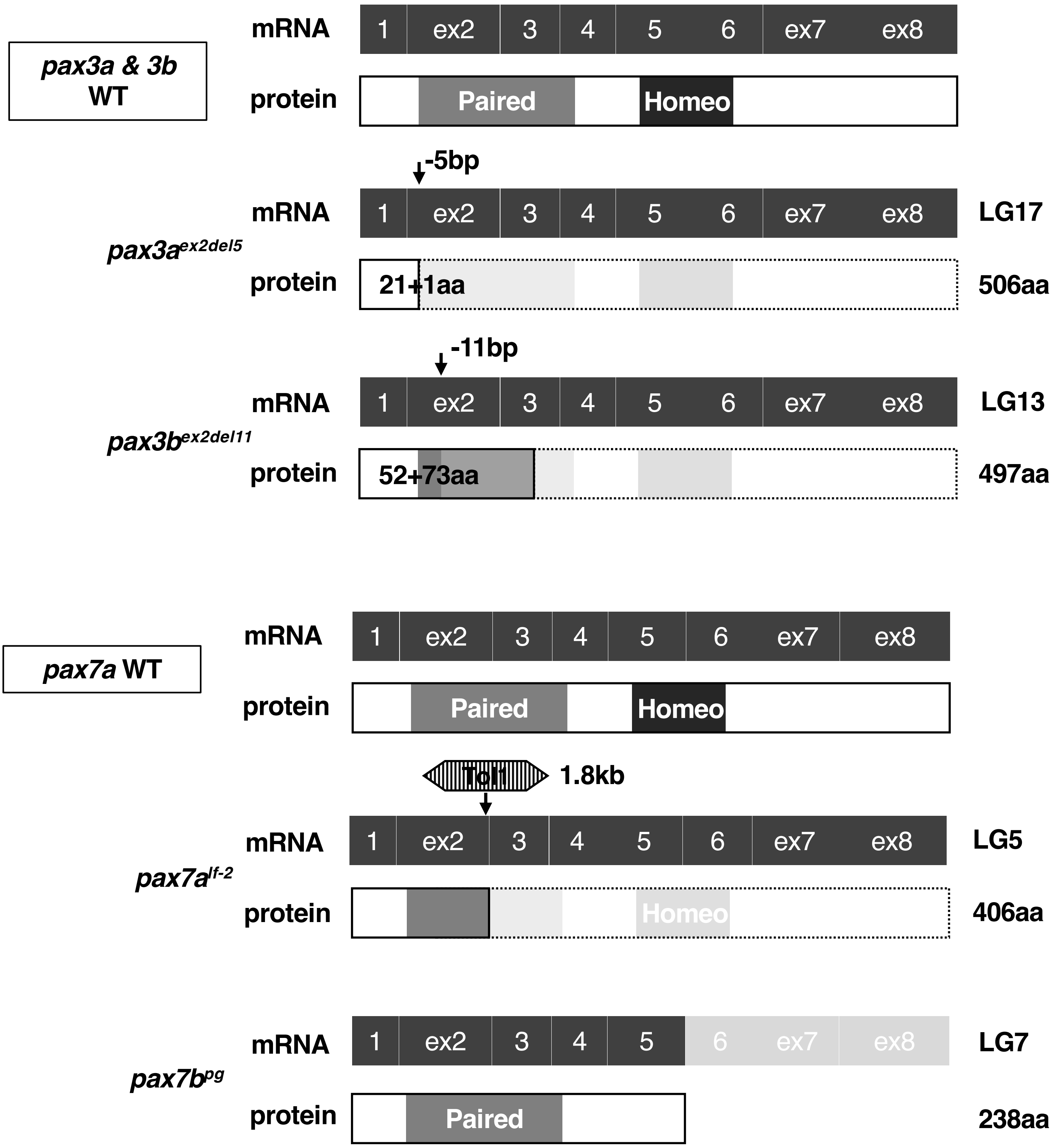

Development • Supplementary information

Fig. S1. The *pax3* and *pax7* mutations in medaka

We generated mutants for *pax3a*, *pax3b* and *pax7b* in medaka using CRISPR/Cas9 or TALEN. We selected a target in the region encoding the paired domain. A five base pair deletion in the second exon of *pax3a* gene (*pax3a^{ex2del5}*) and an eleven base pair deletion in the third exon in *pax3b* gene (*pax3b^{ex2del11}*) were generated in medaka. All the mutants were viable and fertile, and so we successfully established each strain.

Schematics of predicted primary structures are shown for wild type (WT) gene or mRNA and protein and mutant protein of Pax3a, Pax3b, Pax7a and Pax7b. The linkage group (LG) where the gene is located is shown on the right to the mRNA. The size (aa, amino acid) of the WT protein is shown to the right to the protein. The position and size (bp) of the deletion or insertion mutation is indicated by arrow in the corresponding exon. The size of the mutant protein is given as the endogenous amino acid sequence + a de novo sequence following the frame-shift due to the deletion mutation (e.g. 21 + 1aa means that the Pax3a^{ex2del5} protein retains 21 N-terminal amino acids and one de novo amino acid). The *pax7a* mutation is an insertional mutation of Tol2 transposon (1.8kb) at the end of exon 2, previously known as *leucophore free-2* mutant (Kimura et al., 2014). Pax7b was found in the medaka genome database as a pseudogene lacking exons 6-8 (ENSORLG00000023957). None of the mutant proteins has a complete paired box (gray) nor a homeobox (black).

Development • Supplementary information

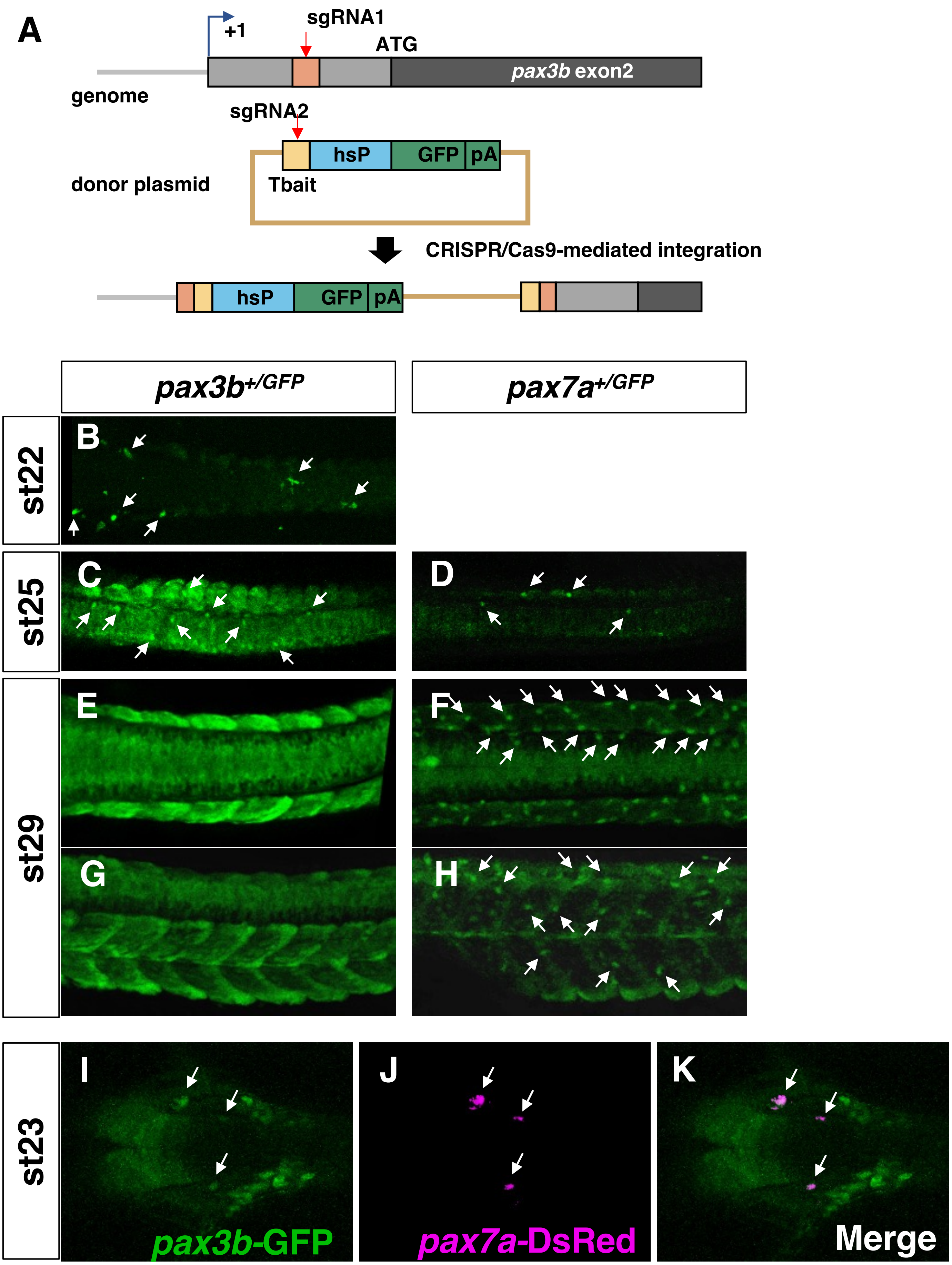

Development • Supplementary information

Fig. S2. Labeling *pax3b*- and *pax7a*-expressing cells in medaka GFP-knock-in transgenic lines

(A) Method for targeted integration of the GFP cDNA using CRISPR/Cas9. sgRNA1 was designed to target a sequence (5'-GGCTAGACAGCAGTGTCCC-3') upstream of the *pax3b* start codon. The sgRNA2 was used to cleave of the donor plasmid containing Tbait sequence upstream of the insertion cassette (hsP-GFP-pA) (Watakabe et al., 2018).

(B, C, E, G, I, K) *pax3b*$^{+/GFP}$ knockin line Tg(*pax3b-hs:GFP*). (D, F, H, J) *pax7a*$^{+/GFP}$ knockin line Tg(*pax7a-hs:GFP*). (J, K) Tg(*pax3b-hs:GFP*); Tg*BAC*(*pax7a-DsRed*). Tg(*pax3b-hs:GFP*) has been established and is reported for the first time in this study. Tg(*pax7a-hs:GFP*) and Tg*BAC*(*pax7a-DsRed*) transgenic lines have been reported previously (Nagao et al., 2018; Watakabe et al., 2018).

The *pax3b*-expressing cells, labeled with GFP fluorescence, are observed in premigratory NCCs, neural tissues and somites at stages 22-25 (B, C). Later than these stages, the fluorescence has disappeared from the NCCs while signals are still observed in the somites and neural tube (E, G). A few cells in premigratory NCCs are positive for fluorescence in the *pax7a*$^{+/GFP}$ embryo at stage 25 (D). The *pax7a*-expressing cells are located in migrating NCCs, expanding ventralaterally (F, H).

The cells that are double positive for GFP and DsRed in The Tg(*pax3b-hs:GFP*); Tg*BAC*(*pax7a-DsRed*) embryo at stage 23 (arrows) indicate that those express both Pax3b and Pax7a. At the same time, the cells positive only for GFP are observed (I, J, K).

Development • Supplementary information

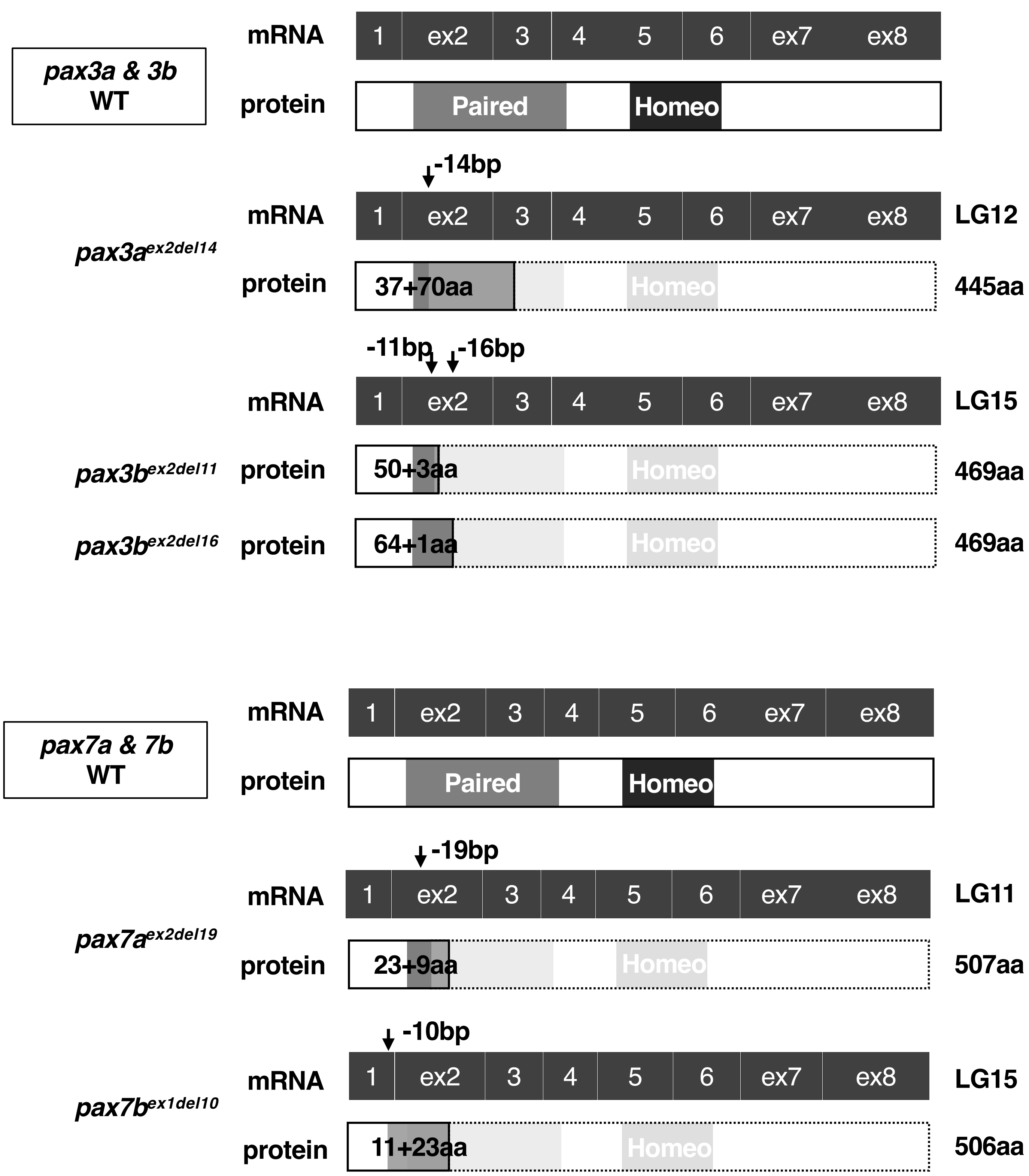

Development • Supplementary information

Fig. S3. The *pax3* and *pax7* mutations in zebrafish

We generated mutants for *pax3a*, *pax3b*, *pax7a* and *pax7b* in zebrafish using CRISPR/Cas9 or TALEN. We selected a target in the region encoding the paired domain. A fourteen base pair deletion in the second exon of *pax3a* gene ($pax3a^{ex2del14}$), an eleven base pair deletion in the second exon of *pax3b* gene ($pax3b^{ex2del11}$), a nineteen base pair deletion in the second exon of *pax7a* gene ($pax7a^{ex2del19}$) and a ten base pair deletion in the first exon of *pax7b* gene ($pax7b^{ex1del10}$) were generated in zebrafish. All the mutants were viable and fertile, and so we successfully established each strain.

In addition, the double mutants of the paralogous genes, *pax3a; pax3b* double and *pax7a; pax7b* double, were also viable and fertile, and were used for our analyses when necessary. Schematics of predicted primary structures are shown for wild type (WT) mRNA and protein and mutant protein of Pax3a, Pax3b, Pax7a and Pax7b. The linkage group (LG) where the gene is located is shown to the right of the mRNA. The size (aa, amino acid) of the WT protein is shown to the right of the protein. The position and size (bp) of the deletion mutation is indicated by arrow in the corresponding exon. The size of the mutant protein is given as the endogenous amino acid sequence + a de novo sequence following the frame-shift due to the deletion mutation (e.g., 37 + 70aa means that $Pax3a^{ex2del14}$ protein retains 37 N-terminal amino acids and 70 de novo amino acids). None of the mutant proteins has a complete paired box (gray) nor a homeobox (black).

Development • Supplementary information

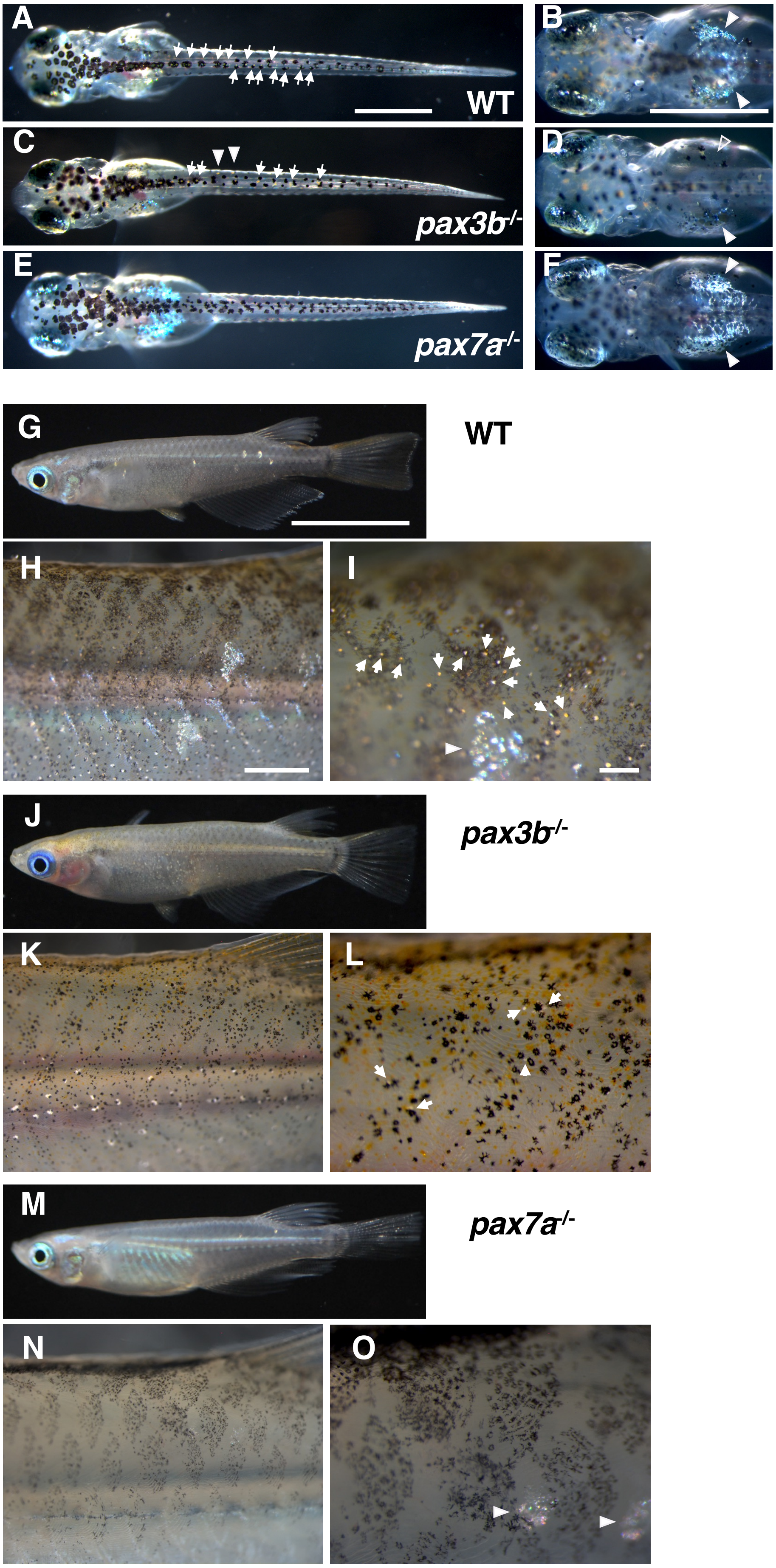

Development • Supplementary information

Fig. S4. Larval and adult gross phenotypes of medaka *pax3b* and *pax7a* mutants (A, C, E) 7 dpf hatchlings. (A) Wild type (WT). (C) *pax3b*$^{-/-}$. (E) *pax7a*$^{-/-}$.
(B, D, F) Close-up views of the dorsal region of the yolk in 7 dpf hatchlings. (B) WT. (D) *pax3b*$^{-/-}$. (F) *pax7a*$^{-/-}$.
(G-O) 4-month-old adult fish. (G, H, I) WT. (J, K, L) *pax3b*$^{-/-}$. (M, N, O) *pax7a*$^{-/-}$. (H, I, K, L, N, O) Close-up views of the anterior region of the dorsal fin of the fish in G, J, and M, respectively.
(A-F) Dorsal views. (G-O) Lateral views.
Scale bars = 0.5 mm in A for A, C, E; 0.5 mm in B for B, D, F; 1 cm in G for G, J, M; 0.15 cm in H for H, K, N; 0.05 cm in I for I, L, O.
Melanophores, leucophores and iridophores seem only slightly reduced in *pax3b*$^{-/-}$ hatchlings (A, C, arrows indicate leucophores; B, D, E, arrowheads indicate iridophores and an open arrowhead is pointing where iridophores are severely reduced). See Fig. 2 B, D, F for xanthophores. These pigment cells appeared to have partially recovered by adulthood (arrows indicate leucophores in I and L), although melanophores look fewer and enlarged, and iridophores might be slightly reduced (compare abdominal iridescence in G, J, M) in the *pax3b* mutant (G-L). In the *pax7a* mutant, leucophores and xanthophores are completely absent at hatching stages (E, see also Fig. 2 F) and during adulthood (M-O). Formation of melanophores and iridophores looks relatively unaffected by loss of Pax7a (E, F, M-O). Arrowheads indicate iridophores in I and O.
As we previously reported (Nagao et al., 2014), it appears that leucophores are distributed more laterally when xanthophores are lost from the adjacent lateral region, as cell-cell interaction seems to determine their relative position. In the case of the *pax3b* mutant (C), leucophores are located more laterally (two arrowheads) where xanthophores should be lost.

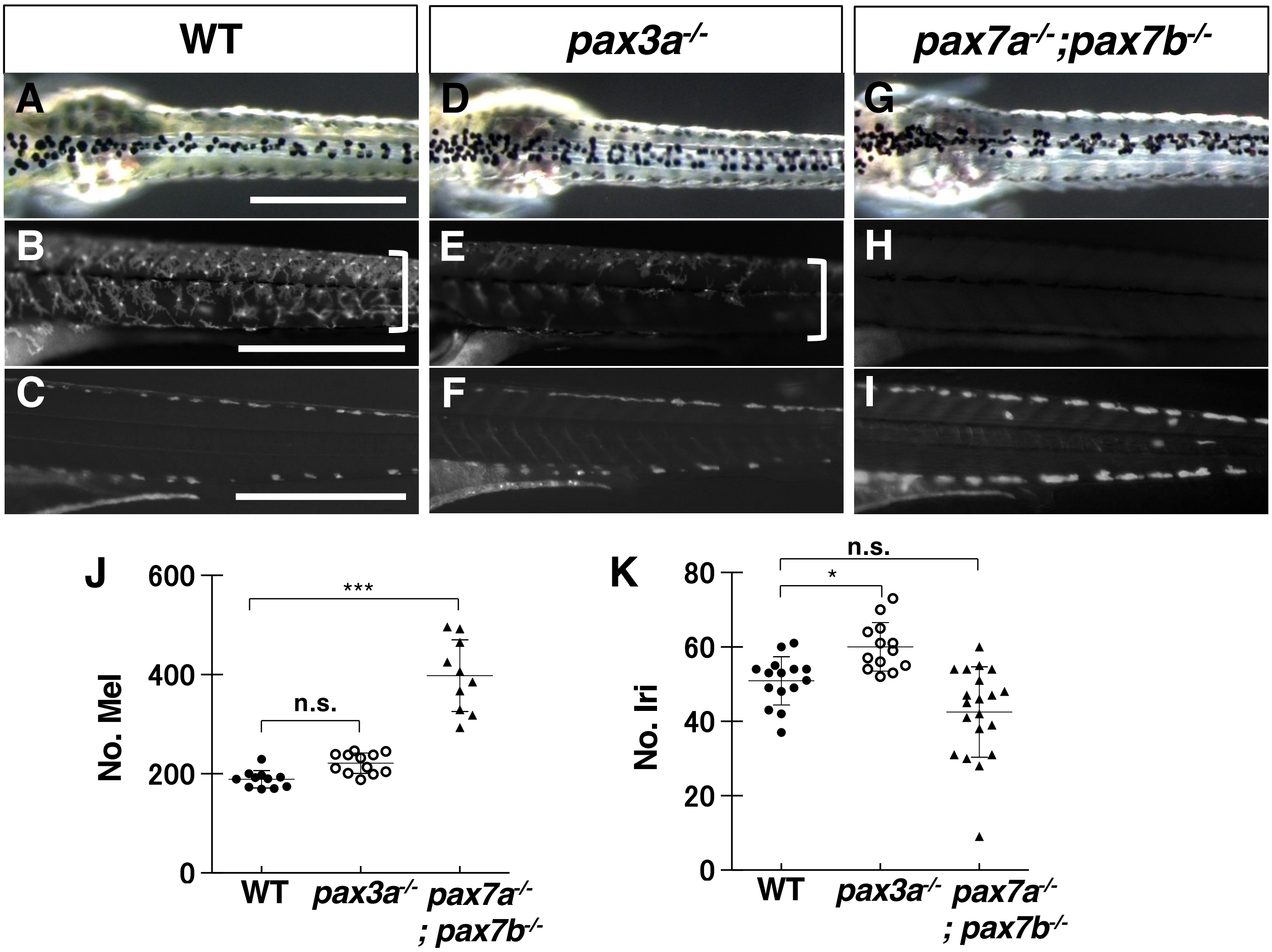

WT
pax3a-/-
pax7a-/-;pax7b-/-
A
B
C
D
E
F
G
H
I
J
K
No. Mel
600
400
200
0
***
n.s.
WT
pax3a-/-
pax7a-/-
; pax7b-/-
No. Iri
80
60
40
20
0
n.s.
*

Fig. S5. Phenotypes of zebrafish *pax3* and *pax7* mutants

(A-I) Zebrafish 4 dpf hatchlings. Pigment cell phenotypes are compared between WT (A, B, C), *pax3a*$^{-/-}$ (D, E, F) and *pax7a*$^{-/-}$; *pax7a*$^{-/-}$ (G, H, I).

(A, D, G) Dorsal views of the trunk in dark field. (B, E, H) Lateral views of the trunk under UV light. (C, F, I) Lateral views of the trunk in dark field. (J, K) Cell counts. Melanophores (J) on the dorsal surface of the trunk are quantified. Iridophores in the dorsal and ventral edges of the trunk are quantified (K).

In zebrafish, as in medaka, xanthophores are severely reduced in *pax3a*$^{-/-}$ mutant hatchlings (xanthophores located in the yellowish part in A and D, and are more prominent in B and E, indicated by square brackets), and completely absent in *pax7a*$^{-/-}$; *pax7a*$^{-/-}$ mutants (G, H), whereas melanophores are unaltered in *pax3a*$^{-/-}$ mutants (A, D, J), while significantly increased in *pax7a*$^{-/-}$; *pax7a*$^{-/-}$ mutants (G, J). Iridophores are slightly but significantly increased in *pax3a*$^{-/-}$ mutants (C, F, K), while not changed in *pax7a*$^{-/-}$; *pax7a*$^{-/-}$ mutants (I, K). Significant difference was determined by Kruscal-Wallis test. ***p<0.05. n. s. = not significant.

Scale bars = 250 µm

Development • Supplementary information

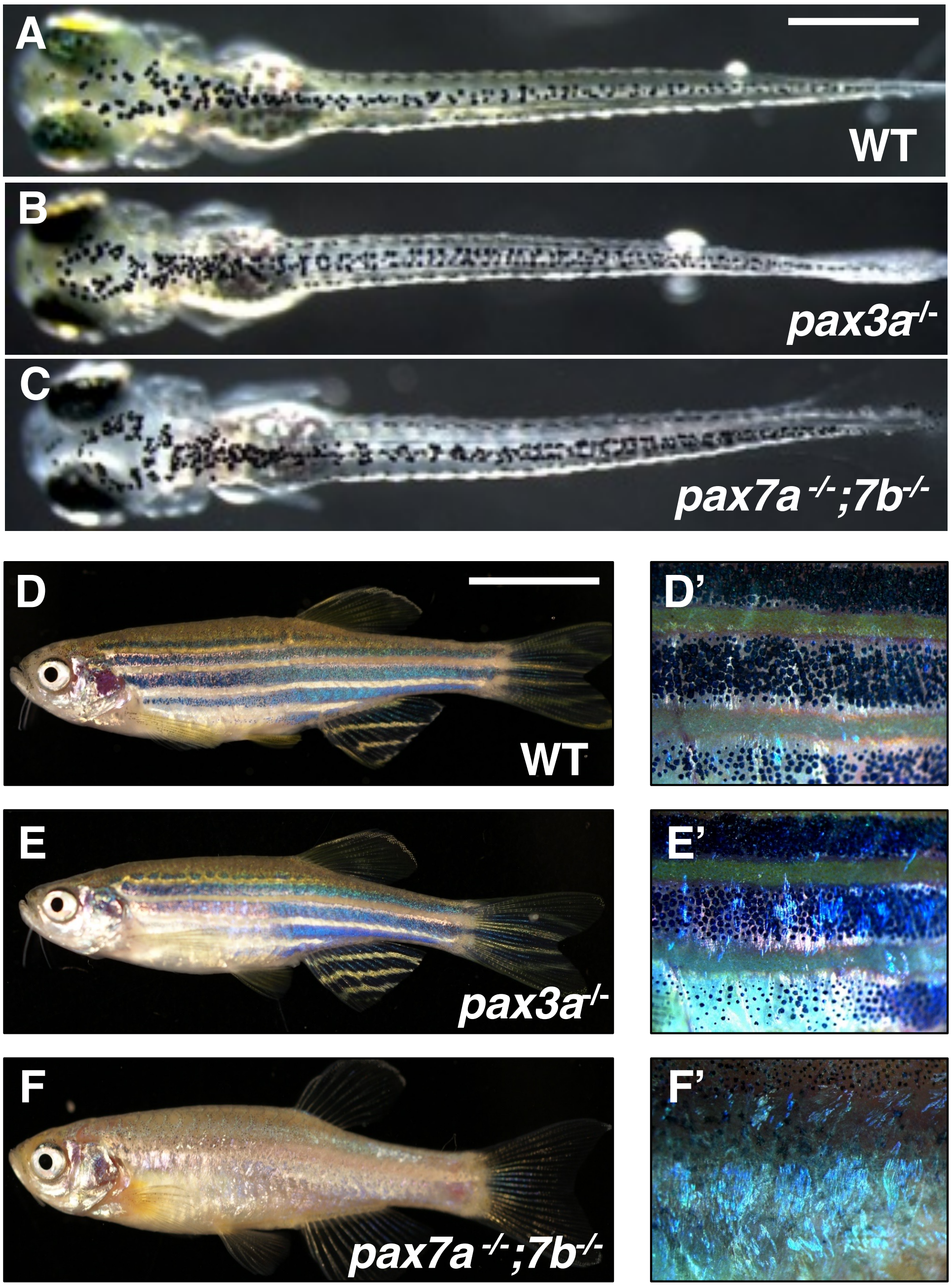
A
WT
B
pax3a-/-
C
pax7a -/-;7b-/-
D
WT
D'
E
pax3a-/-
E'
F
pax7a -/-;7b-/-
F'

Fig. S6. Larval and adult gross phenotypes of zebrafish *pax3a* and *pax7a; pax7b* mutants

(A-C) 4 dpf hatchlings. Dorsal views. Whole images of the same larvae shown in Suppl. Fig. 5. (D-F) 4-month-old adult fish. (A, D) Wild type (WT). (B, E) *pax3a*$^{-/-}$. (C, F) *pax7a*$^{-/-}$; *pax7b*$^{-/-}$. (D', E', F') Close-up images of the skin in D, E, F, respectively. Xanthophores are severely reduced in the *pax3a* mutant hatchling (A, B, see also Suppl. Fig. 5 A, B, D, E), but are restored in the adult (D, D', E, E'). The *pax7a*$^{-/-}$; *pax7b*$^{-/-}$ double mutant is completely defective for xanthophore formation not only at the hatching stage (C, see also Suppl. Fig. 5 G, H) but also at the adult stage (F, F').

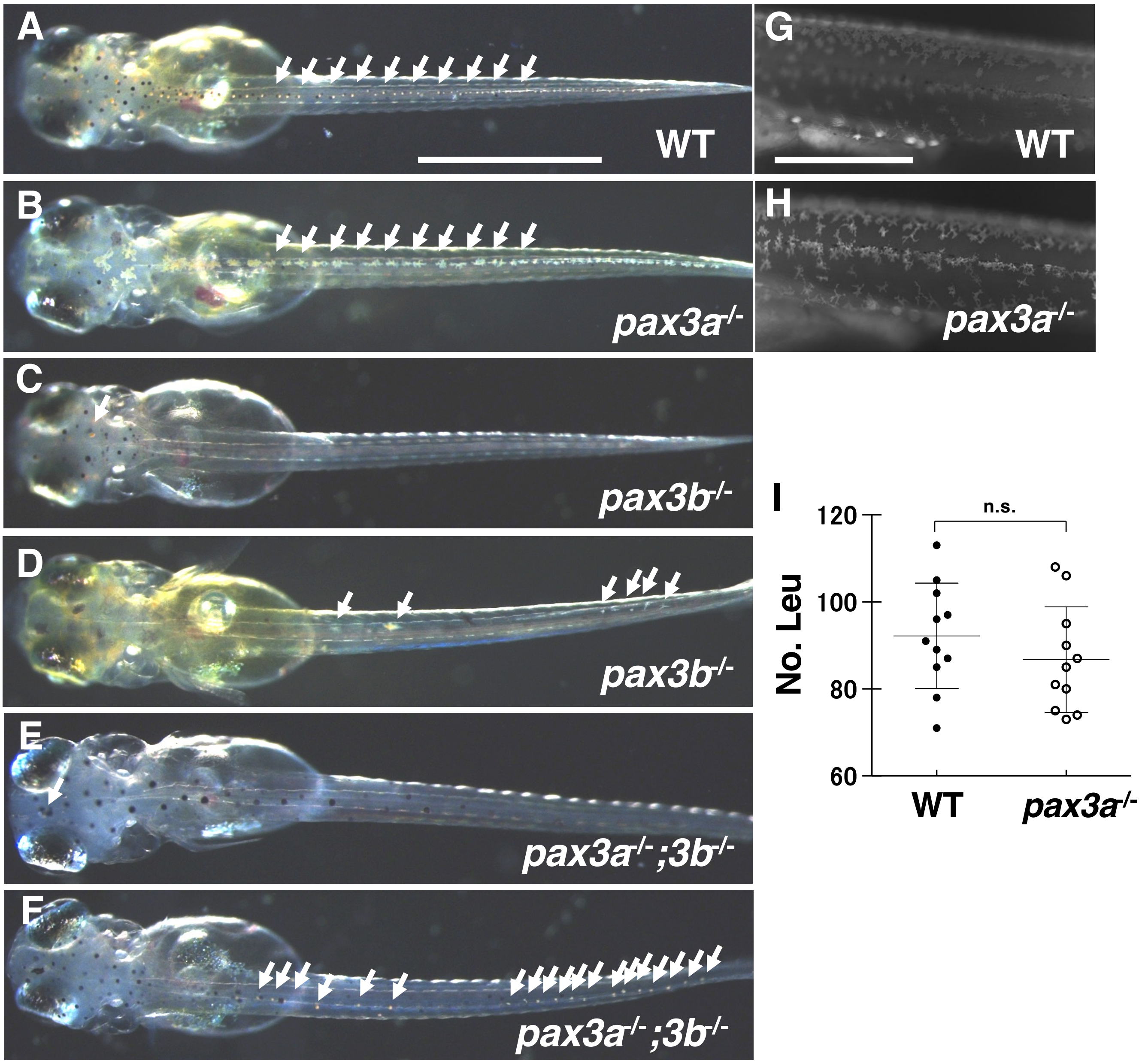
A
WT
B
pax3a-/-
C
pax3b-/-
D
pax3b-/-
E
pax3a-/- ;3b-/-
F
pax3a-/- ;3b-/-
G
WT
H
pax3a-/-
I
n.s.
120
100
80
60
No. Leu
WT
pax3a-/-

Fig. S7. Medaka *pax3* mutant phenotypes

(A-F) 7 dpf hatchlings with d-rR background, in which melanophore pigmentation is severely defective. Dorsal views. Leucophores located in the dorsal midline were compared between WT (A) and *pax3a*$^{-/-}$ (B), *pax3b*$^{-/-}$ (C, D), *pax3a*$^{-/-}$; *pax3b*$^{-/-}$ (E, F) mutants. The *pax3a* mutation does not appear to affect the pigment cell development, nor does it exacerbate the *pax3b* phenotype in the double homozygous *pax3a*$^{-/-}$; *pax3b*$^{-/-}$ hatchling. (G, H) Autofluorescent xanthophores in WT (G) and *pax3a*$^{-/-}$ (H). Lateral views of the trunk under UV light. (I) Leucophore counts on the dorsal body in WT and *pax3a*$^{-/-}$ mutant. The double homozygotes failed to grow to adults. Xanthophore formation is not affected in *pax3a*$^{-/-}$ mutant (G, H). Arrows are pointing at leucophores on the body (A, B, D, F) and on the head (C, E). Scale bar = 0.5 mm (A-F) and 250 µm (G, H)

Development • Supplementary information

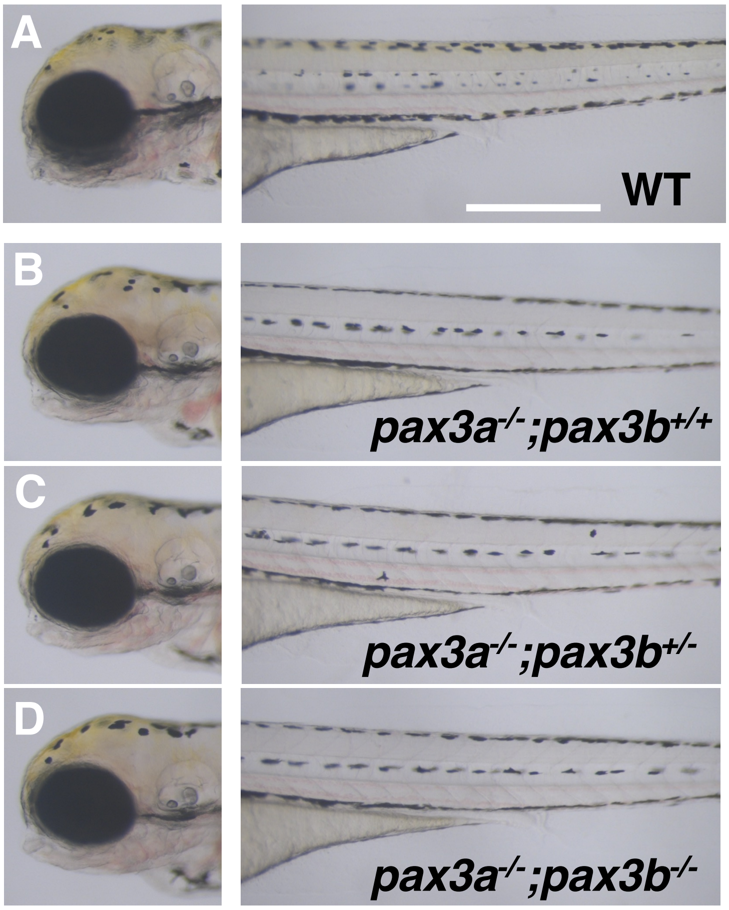

Fig. S8. Zebrafish *pax3* mutant phenotypes

(A-D) 4 dpf hatchlings. Lateral views. Left panels show head region. Right panels show trunk and tail regions. (A) WT. (B) *pax3a*$^{-/-}$. (C) *pax3a*$^{-/-}$, *pax3b*$^{+/-}$. (D) *pax3a*$^{-/-}$; *pax3b*$^{-/-}$. Like medaka *pax3a* mutant, zebrafish *pax3b* homozygotes show no obvious pigment cell phenotypes. The *pax3b* mutation does not appear to exacerbate the *pax3a* phenotype in the *pax3a; pax3b* compound mutant hatchlings (A, B, C, D).

Development • Supplementary information

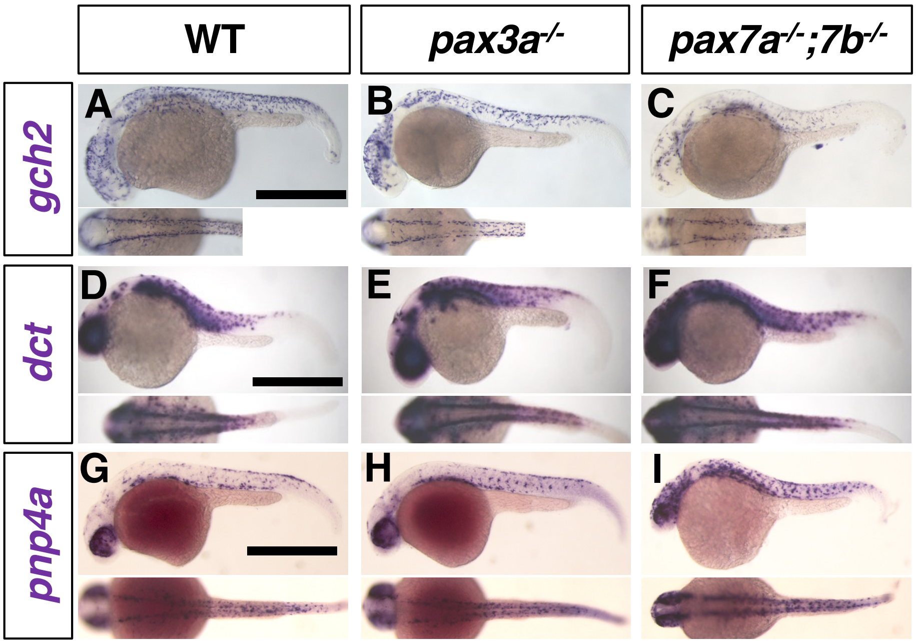

Fig. S9. Pigment cell progenitors in zebrafish *pax3a* and *pax7a; pax7b* mutant embryos

27 hpf. (A-C) *gch2*. (D-F) *dct*. (G-I) *pnp4a*. (A, D, G) WT. (B, E, H) *pax3a*$^{-/-}$ mutant. (C, F, I) *pax7a*$^{-/-}$; *pax7b*$^{-/-}$ mutant. (A-L) Lateral views at top and dorsal views at bottom. Scale bar = 250 µm

The *gch2*-expressing xanthophore progenitors are severely decreased in *pax3a* mutant (A, B) and in *pax7a; pax7b* mutant (C). The *dct*-expressing melanophore progenitors are comparable in WT (D) and in *pax3a* mutant (E), while slightly increased in *pax7a; pax7b* mutant (F). Development of the *pnp4a*-expressing iridophore progenitors is not noticeably altered in the body in *pax3a* mutant (G, H), and unaltered or even increased in *pax7a; pax7b* mutant (I).

Development • Supplementary information

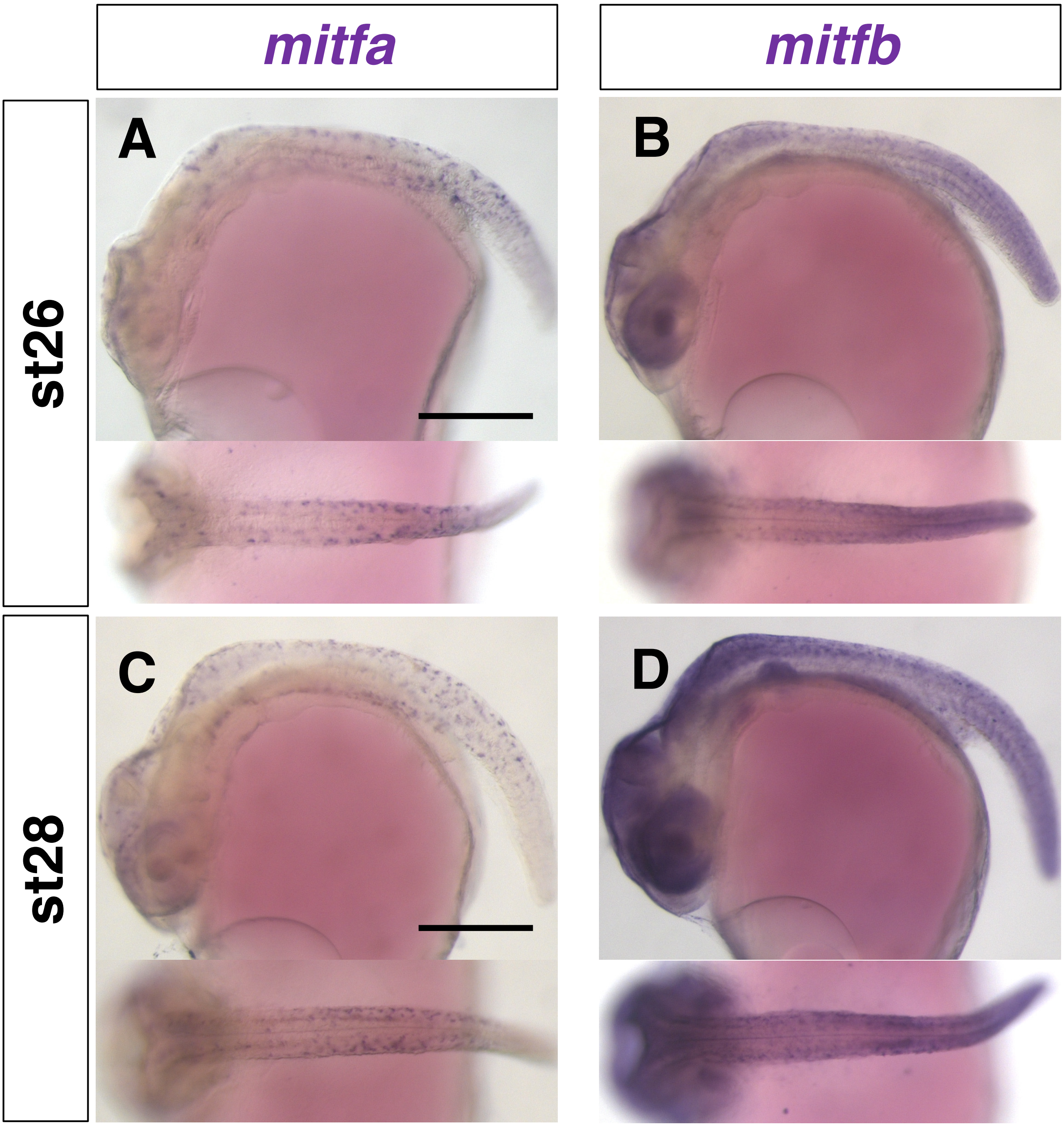

mitfa
mitfb
st26
st28
A
B
C
D

Fig. S10. Comparison of *mitfa* and *mitfb* expression in medaka

(A, C,) *mitfa*. (B, D) *mitfb*. (A, B) Stage 26. (C, D) Stage 28.

Lateral views top. Dorsal views below. All anterior to the left. Scale bars = 250 µm.

In situ analyses were performed with medaka embryos. The *mitfa*-expressing cells are observed in the dorsal and lateral surface of the embryonic body and in the eye region (A, C). Stronger signals of the *mitfb*-expressing cells are detected at locations similar to those of the *mitfa*-expressing cells (B, D). The *mitfa* expression seems to be specific for the NCCs, perhaps pigment cell progenitors, whereas the *mitfb* expression is detected in the NCCs but also in the others, spinal cord, etc.

Pigment cell progenitors with Mitf expression are likely to be detected more clearly with *mitfa* than with *mitfb* as a probe by in situ, which is the reason why we examined *mitfa* rather than *mitfb* in Fig. 5.

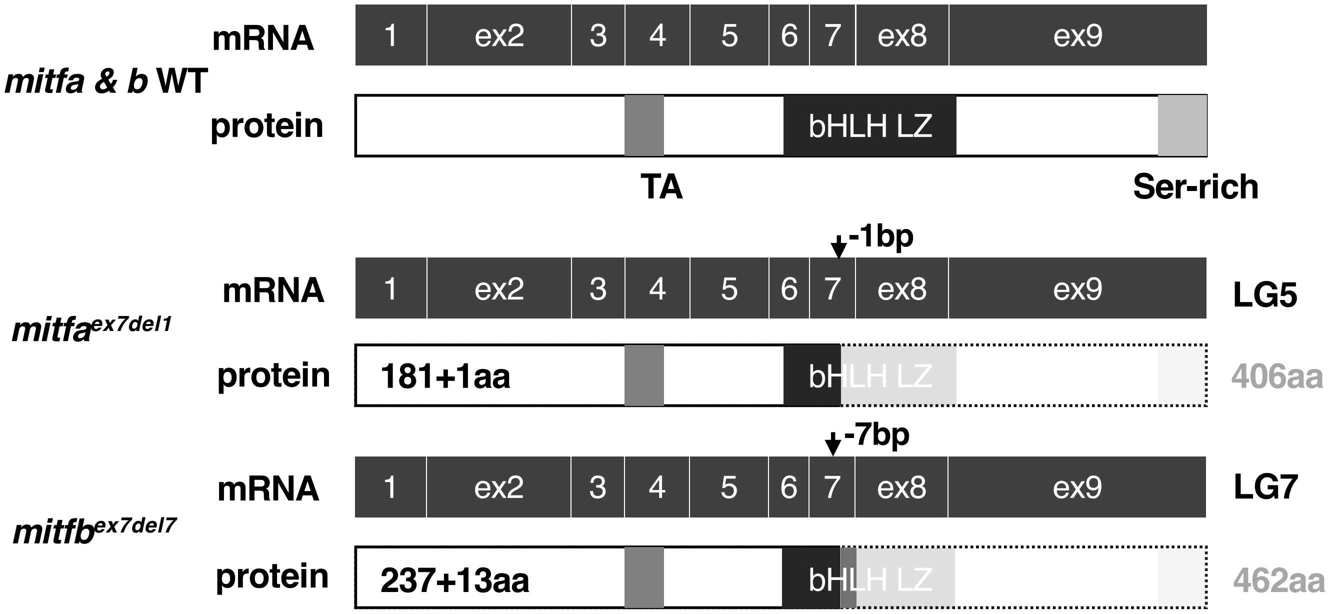

Fig. S11. The *mitfa* and *mitfb* mutations in medaka

We generated mutants for *mitfa* and *mitfb* in medaka by CRISPR/Cas9. Both homozygotes were viable and fertile, and so we successfully established each strain. In addition, the *mitfa; mitfb* double mutants were also viable and fertile, and used for our analyses when necessary.

Schematics of predicted primary structures are shown for wild type (WT) gene or mRNA and protein and mutant protein of Mitfa and Mitfb. The linkage group (LG) where the gene is located is shown on the right to the mRNA. The size (aa, amino acid) of the WT protein is shown right to the protein. The position and size (bp) of the deletion or insertion mutation is indicated by an arrow in the corresponding exon. The size of the mutant protein is given as the endogenous amino acid sequence + a de novo sequence following the frame-shift due to the deletion mutation (e.g., 181 + 1aa means that the Mitfa^{ex7del1} protein retains 181 N-terminal amino acids and one de novo amino acid). The Mitfb^{ex7del7} protein contains 237 N-terminal amino acids and 13 de novo amino acids. Neither of Mitfa and Mitfb proteins has a complete basic helix-loop-helix leucine zipper (bHLH-LZ) domain (black), although both retain the transactivation domain (TA).

Development • Supplementary information

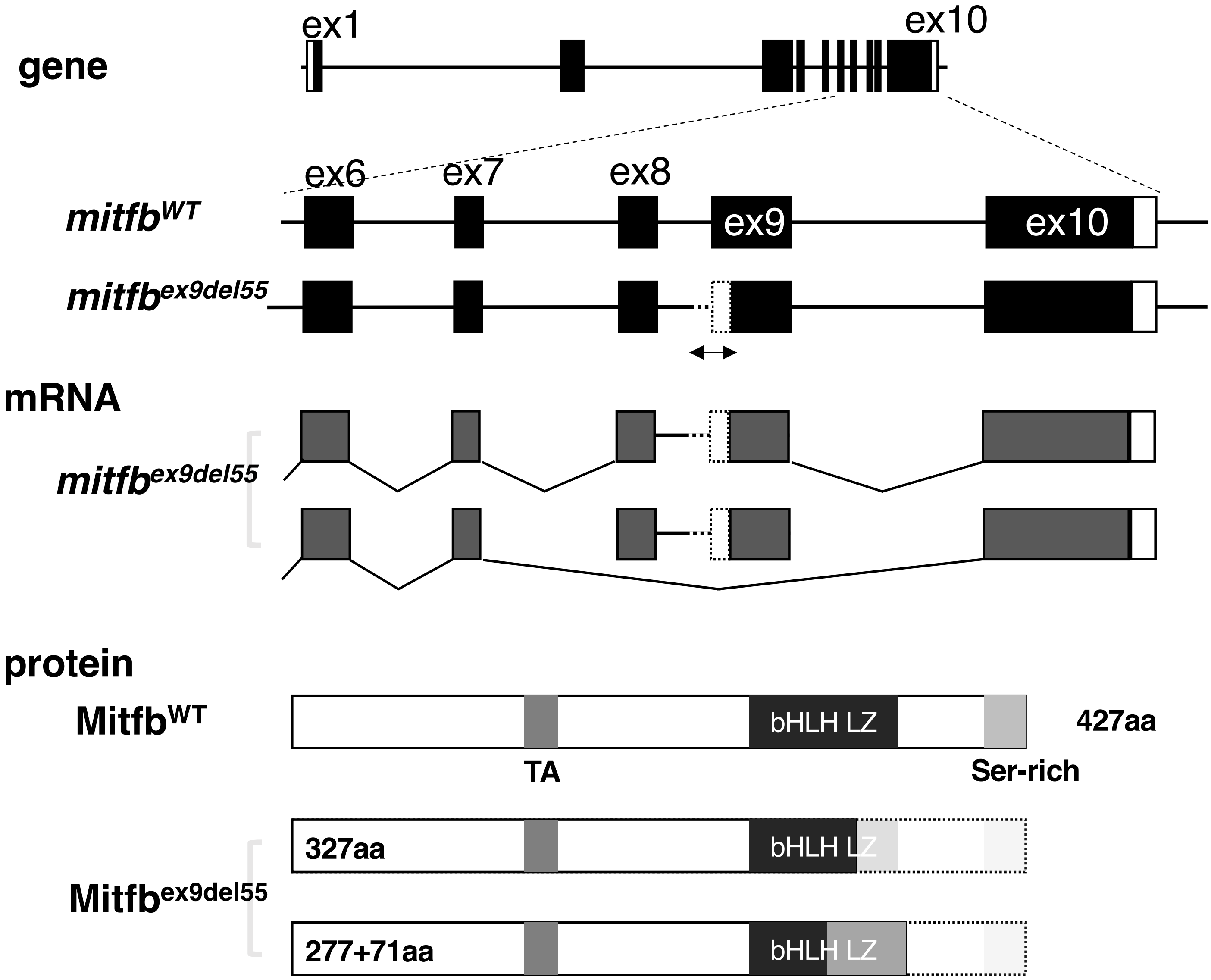

Development • Supplementary information

Fig. S12. The *mitfb* mutation in zebrafish

In order to test if the requirement of Mitfs for xanthophore formation is conserved in zebrafish, we generated an *mitfb* mutation in zebrafish *mitfa*^{nacre} background using CRISPR/Cas9. A guide RNA was designed to target exon 9: 5'-GAACAAGGGCACGATTCTGAAGG-3' (Integrated DNA Technology) encoding the C-terminal half of bHLH domain. A 55 base pair deletion generated (*mitfb*^{ex9del55}) expands the intron 8 and exon 9.

Schematics of primary structures of the gene, mRNA and protein are shown for wild type (WT) and Mitfb^{ex9del55}. RT-PCR and sequencing analyses revealed that the mutant allele produced two alternative mRNAs: one contained the intron 8 sequence between exon 8 and exon 9 but lacked the 55bp deletion sequence. The other mRNA skipped exon 8 and exon 9 with the 3'-end of exon 7 and the 5'-end of exon 10 joined. The mutant proteins have truncated C terminus of bHLH domain: the former mRNA containing intron 8 had a stop codon at the 5'-region of the intron, and the latter encoded de novo amino acid sequence (71 amino acids) after the absence of exon 8 and exon 9. None of the mutant proteins has a complete bHLH (dark gray) or a serine-rich domain (light gray), although both retain the transactivation domain (TA).
