# Supplementary material for: A gene regulatory network combining Pax3/7, Sox10 and Mitf generates diverse pigment cell types in medaka and zebrafish
Source: Development. 2023 Oct 12;150(19):dev202114. doi: 10.1242/dev.202114 (PMC10617610; doi:10.1242/dev.202114)
Supplement: Supplementary file 1 [file develop-150-202114_review_history.pdf]

A gene regulatory network combining Pax3/7, Sox10 and Mitf generates diverse pigment cell types in medaka and zebrafish

Motohiro Miyadai, Hiroyuki Takada, Akiko Shiraishi, Tetsuaki Kimura, Ikuko Watakabe, Hikaru Kobayashi, Yusuke Nagao, Kiyoshi Naruse, Shin-ichi Higashijima, Takashi Shimizu, Robert N. Kelsh, Masahiko Hibi and Hisashi Hashimoto
DOI: 10.1242/dev.202114

Editor: Steve Wilson

Review timeline

Original submission:	23 June 2023
Editorial decision:	18 July 2023
First revision received:	18 August 2023
Accepted:	11 September 2023

Original submission

First decision letter

MS ID#: DEVELOP/2023/202114

MS TITLE: Pax3 and Pax7 function in combination with Mitf to generate melanophores and xanthophores in medaka and zebrafish

AUTHORS: Motohiro Miyadai, Hiroyuki Takada, Akiko Shiraishi, Tetsuaki Kimura, Ikuko Watakabe, Hikaru Kobayashi, Yusuke Nagao, Kiyoshi Naruse, Shin-ichi Higashijima, Takashi Shimizu, Robert Kelsh, Masahiko Hibi, and Hisashi Hashimoto

I have now received all the referees' reports on the above manuscript, and have reached a decision. The referees' comments are appended below, or you can access them online: please go to BenchPress and click on the 'Manuscripts with Decisions' queue in the Author Area.

As you will see, the referees express considerable interest in your work, but have some criticisms and suggestions for improving your manuscript. If you are able to revise the manuscript along the lines suggested, I will be happy receive a revised version of the manuscript. Please note that Development will normally permit only one round of major revision. If it would be helpful, you are welcome to contact us to discuss your revision in greater detail. Please send us a point-by-point response indicating your plans for addressing the referee’s comments, and we will look over this and provide further guidance.

Please attend to all of the reviewers' comments and ensure that you clearly highlight all changes made in the revised manuscript. Please avoid using 'Tracked changes' in Word files as these are lost in PDF conversion. I should be grateful if you would also provide a point-by-point response detailing how you have dealt with the points raised by the reviewers in the 'Response to Reviewers' box. If you do not agree with any of their criticisms or suggestions please explain clearly why this is so.

© 2023. Published by The Company of Biologists under the terms of the Creative Commons Attribution License (https://creativecommons.org/licenses/by/4.0/).

Reviewer 1

Advance summary and potential significance to field

This paper assesses the roles of Pax3 paralogues, Pax7 paralogues, and Mitf paralogues in the development of xanthophores in medaka and zebrafish. Because it covers many mutants in two species it can be a bit hard to follow, but the authors keep the story as clear as possible. Together the loss of function and gain of function studies in medaka support the model that Pax7 co-operates with Mitf to promote the xanthophore lineage while inhibiting Mitf's ability to promote the melanophore lineage. The zebrafish results are consistent except for the surprising finding that xanthophores are still present in mitfa/mitfb double mutants. This unexpected finding is explained by the possibility that Tfec compensates for the loss of Mitf in zebrafish —which is unsatisfying because why would this not occur in medaka?

Nonetheless, the model from medaka is a nice extension of earlier work from mouse showing that Pax3 can inhibit MITF activity at the DCT promoter. I have no major criticisms of the study.

Comments for the author

Minor:
An important result, the phenotype of zebrafish mitfa/mitfb double mutants, is presented for the first time in the Discussion section, which is non-standard and inappropriate. This result should be moved to the Results section.

Line 218: typo
“and ambiguous in both medaka and zebrafish (Suppl. Figs. 4 B, D, F, 6 C, F, I, K) “
Underlined number should be 5, not 6.

Line 238: word choice
“In medaka, expression of gch in xanthophore/leucophore progenitors were severely lost “
Underlined line should be “reduced”. Lost is a binary quality, it cannot be modified.
Line 170 and many other places: It is easier on the reader if figure citations are present before the description of the figure, not after. This is especially important when the figure citation refers to multiple panels.

Original:
“In situ analyses showed that pax3b-expressing cells were observed in dorsal parts of the midbrain hindbrain and spinal cord, dorsal somites and premigratory NCCs at early somite stages (Fig. 1A-A’’). “

Suggestion:
“In situ analyses showed that (Fig. 1A-A’’) pax3b-expressing cells were observed in dorsal parts of the midbrain, hindbrain and spinal cord, dorsal somites and premigratory NCCs at early somite stages”

Line 272 Original:
“The mitfa-expressing cells were severely reduced in stage 28 medaka pax3b mutants, whereas comparable or rather increased in pax7a mutant compared to wild-type embryos (Fig. 5 A, B, C).

Suggest
“The mitfa-expressing cells were severely reduced in stage 28 medaka (Fig. 5B) pax3b mutants, whereas comparable or rather increased in (Fig. 5C) pax7a mutant compared to wild-type embryos (Fig. 5 A).”

or

“In comparison to in stage 28 medaka (Fig. 5A) wild-type embryos, the mitfa-expressing cells were severely reduced in (Fig. 5B) pax3b mutants, whereas comparable or rather increased in (Fig. 5C) pax7a mutant embryos.”

© 2023. Published by The Company of Biologists under the terms of the Creative Commons Attribution License (https://creativecommons.org/licenses/by/4.0/).

Reviewer 2

Advance summary and potential significance to field

This manuscript explores the gene-regulatory network (GRN) underlying the development of pigment cells in fish. There is a simple GRN for the single pigment cell type in mammals, the melanocyte, comprising PAX3 and SOX10 jointly activating the "master regulator" gene MITF. Melanocyte development in mice has long been an simple exemplar for regulation of a developmental process. Fish have 3 (zebrafish) or 4 (Medaka) pigment cell types, originating from the same progenitor neural crest cell (NCC) population. Arguably the GRN for fish pigment cells is a more complex but still relatively tractable model for developmental processes.

Comments for the author

This manuscript explores the gene-regulatory network (GRN) underlying the development of pigment cells in fish. There is a simple GRN for the single pigment cell type in mammals, the melanocyte, comprising PAX3 and SOX10 jointly activating the "master regulator" gene MITF. Melanocyte development in mice has long been an simple exemplar for regulation of a developmental process. Fish have 3 (zebrafish) or 4 (Medaka) pigment cell types, originating from the same progenitor neural crest cell (NCC) population. Arguably the GRN for fish pigment cells is a more complex but still relatively tractable model for developmental processes. Fish have the additional complication of having a partially duplicated genome relative to mammals, so there are 2 copies of each of the 3 genes studied in mammals (although one of the two pax7 genes appears to be a pseudogene harboring a partial deletion.

This manuscript describes a comprehensive and well executed study of SOX10, PAX3 and MITF in Medaka and zebrafish examining pigment cell phenotype and gene expression in wild type fish and in fish with mutations in these genes. In addition, key experiments are described where ectopic expression of MITF is found in response to injection of PAX3 and SOX10 together, but not separately.

Furthermore, MITF injection results in ectopic expression of markers of both melanophores and xanthophores. Coinjection of PAX7 represses this induction. The GRN in Figure 9 is an good representation of the findings of this manuscript along with previous work.

Minor points
where clarification would be useful:
There are differences between species in effect of paralog mutation, so pax3a mutants in zebrafish are more like pax3b mutants in Medaka. If I understand correctly, Medaka requires mutation of both mitfa and b to produce the same phenotype as mitfa alone in zebrafish. Can the authors comment? Is zebrafish pax3a truly orthologous to pax3a in Medaka?
In lines 193 to 194, they say that because there are cells that express pax3a alone even after pax7 is active this means that pax3b positive cells are in another lineage. This should be more carefully phrased. It is possible that these cells are still in a pre-pax7 expression state.
In lines 335 to 341, they say that mitfb but not mitfa plays a crucial role in melanophore development in medaka. However the dounle mutant is much more severe than the single mitfb mutation, indicating some redundancy. Surely mitfa is playing some crucial role?
Ectopic expression of Mitf can be induced by injection of Pax3 and Sox10 together, but by neither alone. However, whilst Pax3 mutants reduce the number of cells expressing mitf in both species, these cells nevertheless do so in absence of pax3. Can the authors comment?

Reviewer 3

Advance summary and potential significance to field

In this work, Motohiro Miyadai et al. explore the role of key pigment cell transcription factors in medaka and zebrafish. The core gene regulatory network Sox10/Pax3/Mitf that regulates

© 2023. Published by The Company of Biologists under the terms of the Creative Commons Attribution License (https://creativecommons.org/licenses/by/4.0/).

melanophore development is conserved from fish to mammals, and also essential for the development of other pigment cell types in fish, e.g. yellow xanthophore and white leucophore. They first analyze the spatial and temporal expression of pax3 and its paralog pax7 by in situ hybridization and transgenic reporter lines. They discovered pax3 is co-expressed with pax7 in NCCs with pax3 expressed prior to pax7. More importantly, pax3 functions upstream to pax7 and sequentially to activate pigment cell master regulator mitfa to induce downstream genes, e.g. melanophore marker dct and xanthophore marker gch. By ectopic RNA expression, pax3 jointly activate mitfa with sox10 which again demonstrates its a very conserved gene regulatory network across vertebrates. The other intriguing question is whether this GRN regulates other types of pigment cells in teleost. They test this nicely by generating and analyzing pax3 mutants pax7 mutants and mitf mutants. These mutants exhibit xanthophore and leucophore defects to different degrees, suggesting previously neglect roles of pax3 and mitf on xanthophore and leucophore development. The authors propose a tripotent model of pigment cell differentiation, in which pax7 serves as a cell fate switch to determine melanophore fate vs. xanthophore fate through mitf. The concept is new and sheds light on the complex functions of mitf. The overall data quality is high. In situ hybridization for gene expression patterns are beautiful. Mutant analyses are well documented across different stages. Functional analyses by synthetic RNA ectopic expression and in situ hybridization are neat and convincing. The conclusions are well-supported by the experiments and will provide important insights to the field.

Comments for the author

1. The title seems misleading, as it could suggest that pax3 and pax7 work together with mitf to generate melanophores and to generate xanthophores. First pax3 mutants don't have obvious melanophore defects. Second, there's no functional analysis to test whether pax3 generates melanophore through mitf, nor how pax3 and pax7 combined to act. Though mitfa expression seems to be delayed in pax3 mutants and perhaps compensation by other factors or conflicting actions from pax3 and pax7, these possibilities requires experimental evidence.
2. The authors explained the mild xanthophore defects in pax3 mutants might result from partial compensation by pax7 at early stage. However, pax7 expression is reduced in pax3 mutants at early stage (Fig 4), which conflicts with their pax7 compensation hypothesis.
3. The conclusion pax7 serves as a cell fate switch that determines melanophore lineage or xanthophore/leucophore lineage is critical to the model they proposed.
The ectopic expression of pax7a promoting xanthophore fate in the presence of mitfa is convincing. However, I think the cellular evidence is not sufficient. The phenotype of pax7a mutants could result from xanthophore-melanophore interactions or xanthophore-melanophore competition in the progenitor pool instead of genetic regulation and fate decision. Lineage tracing the cell differentiation of mitfa+ pax7- cells vs. mitfa+ pax7+ cells would be a straightforward approach and necessary to support the model.
4. I appreciate the authors' effort to investigate the mechanisms in parallel in medaka and zebrafish, which provides a broader picture to the audience. In the current manuscript, such comparative analysis indeed emphasizes the overall conservation of this network between teleosts. Yet an intriguing difference between the two species is that xanthophores are totally lost in medaka mitf mutant but not zebrafish mitf mutant. The authors didn't look further into this and only speculated that tfec might compensate for the loss of mitf in zebrafish.
To address it by comparing tfec compensatory expression and function between medaka and zebrafish would strengthen the model, and promote the work to a better example of cross species comparative analysis.

Minor issues:
1. The authors claim leucophores only exist in medaka but not zebrafish. In fact there are leucophores in adult zebrafish as well.
2. In Fig. 2A, arrows indicate 3 yellow cells and 1 white cell. The audience might be confused according to the description in the introduction, xanthophores are yellow and leucophores are white. In B, C and D, some arrows are pointing to blurry fluorescent cells. Could you find better examples to avoid the blurriness which would affect counting? There's no description for leucophores having fluorescent in the manuscript. In addition, there are similar ventral bright iridophores that could be mistaken as leucophores. I think additional descriptions would help readers to distinguish different cell types.

© 2023. Published by The Company of Biologists under the terms of the Creative Commons Attribution License (https://creativecommons.org/licenses/by/4.0/).

3. Suppl. Fig. 4 Leucophores are dispersed and distributed laterally in pax3b mutants compared to WT. Does pax3b mutation also affect leucophore morphology and patterning, or is it just individual variation? It's hard to compare xanthophore leucophore and iridophore in the close ups H, J and L.
4. Suppl. Fig. 5 C, F and I look like green fluorescent channel rather than dark field. The example of pax3a mutants in D seems to have many more melanophores than WT, similarly in Suppl. Fig. 6B.
5. Fig. 5 G-I in the figure legend but not in the figure.
6. Fig. 6 There's a white scale bar covered by the label gch.

First revision

Author response to reviewers' comments

Response to Reviewers' Comments'

Reviewer 1 Advance Summary and Potential Significance to Field:
This paper assesses the roles of Pax3 paralogues, Pax7 paralogues, and Mitf paralogues in the development of xanthophores in medaka and zebrafish. Because it covers many mutants in two species it can be a bit hard to follow, but the authors keep the story as clear as possible. Together the loss of function and gain of function studies in medaka support the model that Pax7 co-operates with Mitf to promote the xanthophore lineage while inhibiting Mitf's ability to promote the melanophore lineage. The zebrafish results are consistent except for the surprising finding that xanthophores are still present in mitfa/mitfb double mutants. This unexpected finding is explained by the possibility that Tfec compensates for the loss of Mitf in zebrafish – which is unsatisfying because why would this not occur in medaka? Nonetheless, the model from medaka is a nice extension of earlier work from mouse showing that Pax3 can inhibit MITF activity at the DCT promoter. I have no major criticisms of the study.

Reviewer 1 Comments for the Author:

Minor:

An important result, the phenotype of zebrafish mitfa/mitfb double mutants, is presented for the first time in the Discussion section, which is non-standard and inappropriate. This result should be moved to the Results section.

Thank you for your suggestion. We have moved the result of zebrafish *mitfa;mitfb* double mutant phenotypes (Suppl. Fig. 13) to the Results section (new Fig. 10).
The sentences have been moved from the figure legend to the Results section in lines 371-380. Numbering of the figures has also been changed accordingly.

Line 218: typo
"and ambiguous in both medaka and zebrafish (Suppl. Figs. 4 B, D, F, 6 C, F, I, K) "
Underlined number should be 5, not 6.

Thank you for pointing out the mistake. We have corrected the figure number (6 to 5) in line 224.

Line 238: word choice
"In medaka, expression of gch in xanthophore/leucophore progenitors were severely lost "
Underlined line should be "reduced". Lost is a binary quality, it cannot be modified.

Thank you for your kind suggestion for an accurate description. We have changed the word 'lost' to 'reduced'.
We found similar cases in the description of *pax7* expression in the *pax3* mutant. We have also changed 'lost' to 'reduced' in appropriate places (lines 265-267, 883, 886)

© 2023. Published by The Company of Biologists under the terms of the Creative Commons Attribution License (https://creativecommons.org/licenses/by/4.0/).

Line 170 and many other places: It is easier on the reader if figure citations are present before the description of the figure, not after. This is especially important when the figure citation refers to multiple panels.

Original:
“In situ analyses showed that pax3b-expressing cells were observed in dorsal parts of the midbrain, hindbrain and spinal cord, dorsal somites and premigratory NCCs at early somite stages (Fig. 1A-A’’).“

Suggestion:
“In situ analyses showed that (Fig. 1A-A’’) pax3b-expressing cells were observed in dorsal parts of the midbrain, hindbrain and spinal cord, dorsal somites and premigratory NCCs at early somite stages”

Line 272

Original:
“The mitfa-expressing cells were severely reduced in stage 28 medaka pax3b mutants, whereas comparable or rather increased in pax7a mutant compared to wild-type embryos (Fig. 5 A, B, C).

Suggest
“The mitfa-expressing cells were severely reduced in stage 28 medaka (Fig. 5B) pax3b mutants, whereas comparable or rather increased in (Fig. 5C) pax7a mutant compared to wild-type embryos (Fig. 5 A).”

or

“In comparison to in stage 28 medaka (Fig. 5A) wild-type embryos, the mitfa-expressing cells were severely reduced in (Fig. 5B) pax3b mutants, whereas comparable or rather increased in (Fig. 5C) pax7a mutant embryos.”
Thank you for your comments. We have changed the position of the figure citations so that they appear before the description of the figures. The changes we have made are:
line 174
lines 179-180
line 192-193
lines 205-206
lines 211-213
line 218
lines 245-251
lines 280-284
lines 300-301
lines 346-351

Reviewer 2 Advance Summary and Potential Significance to Field:

This manuscript explores the gene-regulatory network (GRN) underlying the development of pigment cells in fish. There is a simple GRN for the single pigment cell type in mammals, the melanocyte, comprising PAX3 and SOX10 jointly activating the "master regulator" gene MITF. Melanocyte development in mice has long been an simple exemplar for regulation of a developmental process. Fish have 3 (zebrafish) or 4 (Medaka) pigment cell types, originating from the same progenitor neural crest cell (NCC) population. Arguably the GRN for fish pigment cells is a more complex but still relatively tractable model for developmental processes.

Reviewer 2 Comments for the Author:
This manuscript explores the gene-regulatory network (GRN) underlying the development of pigment cells in fish. There is a simple GRN for the single pigment cell type in mammals, the melanocyte, comprising PAX3 and SOX10 jointly activating the "master regulator" gene MITF. Melanocyte development in mice has long been an simple exemplar for regulation of a

© 2023. Published by The Company of Biologists under the terms of the Creative Commons Attribution License (https://creativecommons.org/licenses/by/4.0/).

developmental process. Fish have 3 (zebrafish) or 4 (Medaka) pigment cell types, originating from the same progenitor neural crest cell (NCC) population. Arguably the GRN for fish pigment cells is a more complex but still relatively tractable model for developmental processes. Fish have the additional complication of having a partially duplicated genome relative to mammals, so there are 2 copies of each of the 3 genes studied in mammals (although one of the two pax7 genes appears to be a pseudogene harboring a partial deletion.

This manuscript describes a comprehensive and well executed study of SOX10, PAX3 and MITF in Medaka and zebrafish examining pigment cell phenotype and gene expression in wild type fish and in fish with mutations in these genes. In addition, key experiments are described where ectopic expression of MITF is found in response to injection of PAX3 and SOX10 together, but not separately.
Furthermore, MITF injection results in ectopic expression of markers of both melanophores and xanthophores. Coinjection of PAX7 represses this induction. The GRN in Figure 9 is an good representation of the findings of this manuscript along with previous work.

Minor points where clarification would be useful:
There are differences between species in effect of paralog mutation, so pax3a mutants in zebrafish are more like pax3b mutants in Medaka. If I understand correctly, Medaka requires mutation of both mitfa and b to produce the same phenotype as mitfa alone in zebrafish. Can the authors comment? Is zebrafish pax3a truly orthologous to pax3a in Medaka?

Thank you for these important points. As mentioned, in medaka both *mitfa* and *mitfb* need to be mutated to produce the same phenotype as *mitfa* alone in zebrafish, in the sense that the mutation(s) cause the defect in melanophore formation. There is also a striking difference between the two species: Loss of Mitfs also results in defects in xanthophore and leucophore formation in medaka, whereas loss of Mitfa only results in a reduction or delay in xanthophore formation in zebrafish. This is discussed in the Discussion section, lines 502-518.
Phylogenetic analysis suggests that zebrafish *pax3a* and medaka *pax3a* are orthologous, but that medaka *pax3b* is more closely related to zebrafish *pax3a* than to zebrafish *pax3b* (i.e. *pax3b* appears to have been lost and *pax3a* duplicated in the medaka lineage). Our experimental data suggest that zebrafish *pax3a* corresponds to medaka *pax3b* in terms of physiological function in pigment cell development. We have not discussed these points so as not to confuse the story.

In lines 193 to 194, they say that because there are cells that express pax3a alone even after pax7 is active this means that pax3b positive cells are in another lineage. This should be more carefully phrased. It is possible that these cells are still in a pre-pax7 expression state.

We totally agree with the reviewer. We have added a sentence to mention a possibility that the *pax3b*-GFP positive but *pax7a*-DsRed negative cells are still in a pre-*pax7* expression state.
The change has been made in lines 198-199, by adding a possibility that the reviewer suggested, 'We observed *pax3b*-GFP positive but *pax7a*-DsRed negative cells, which may be cells that have not yet reached the state where they are ready to express *pax7a*, but which may indicate that *pax3b*-expressing cells give rise to pigment cell populations other than *pax7a*-expressing xanthophore/leucophore progenitors.'.

In lines 335 to 341, they say that mitfb but not mitfa plays a crucial role in melanophore development in medaka. However the dounle mutant is much more severe than the single mitfb mutation, indicating some redundancy. Surely mitfa is playing some crucial role?
Thank you for your kind suggestion. We have replaced the word 'crucial' with 'more central' in line 348, and added 'together' in line 353. As pointed out, Mitfs play a crucial role, but not a single Mitf, either Mitfa or Mitfb, is crucial.

Ectopic expression of Mitf can be induced by injection of Pax3 and Sox10 together, but by neither alone. However, whilst Pax3 mutants reduce the number of cells expressing mitf in both species, these cells nevertheless do so in absence of pax3. Can the authors comment?
Thank you for commenting on an important point. We have no direct experimental evidence to explain the observation, but as we discuss in the paper (lines 403-411 and 422-427), we speculate that other factor(s), perhaps expressed transiently, partially compensate for Pax3 to activate *mitf*

© 2023. Published by The Company of Biologists under the terms of the Creative Commons Attribution License (https://creativecommons.org/licenses/by/4.0/).

expression. For example, the expression of *pax7* may be only partially dependent on *pax3*, so residual *pax7* could partially compensate for the loss of *pax3*.

Reviewer 3 Advance Summary and Potential Significance to Field:
In this work, Motohiro Miyadai et al. explore the role of key pigment cell transcription factors in medaka and zebrafish. The core gene regulatory network Sox10/Pax3/Mitf that regulates melanophore development is conserved from fish to mammals, and also essential for the development of other pigment cell types in fish, e.g. yellow xanthophore and white leucophore. They first analyze the spatial and temporal expression of pax3 and its paralog pax7 by in situ hybridization and transgenic reporter lines. They discovered pax3 is co-expressed with pax7 in NCCs with pax3 expressed prior to pax7. More importantly, pax3 functions upstream to pax7 and sequentially to activate pigment cell master regulator mitfa to induce downstream genes, e.g. melanophore marker dct and xanthophore marker gch. By ectopic RNA expression, pax3 jointly activate mitfa with sox10 which again demonstrates its a very conserved gene regulatory network across vertebrates. The other intriguing question is whether this GRN regulates other types of pigment cells in teleost. They test this nicely by generating and analyzing pax3 mutants, pax7 mutants and mitf mutants. These mutants exhibit xanthophore and leucophore defects to different degrees, suggesting previously neglect roles of pax3 and mitf on xanthophore and leucophore development. The authors propose a tripotent model of pigment cell differentiation, in which pax7 serves as a cell fate switch to determine melanophore fate vs. xanthophore fate through mitf. The concept is new and sheds light on the complex functions of mitf. The overall data quality is high. In situ hybridization for gene expression patterns are beautiful. Mutant analyses are well documented across different stages. Functional analyses by synthetic RNA ectopic expression and in situ hybridization are neat and convincing. The conclusions are well-supported by the experiments and will provide important insights to the field.

Reviewer 3 Comments for the Author:
1. The title seems misleading, as it could suggest that pax3 and pax7 work together with mitf to generate melanophores and to generate xanthophores. First, pax3 mutants don’t have obvious melanophore defects. Second, there’s no functional analysis to test whether pax3 generates melanophore through mitf, nor how pax3 and pax7 combined to act. Though mitfa expression seems to be delayed in pax3 mutants and perhaps compensation by other factors or conflicting actions from pax3 and pax7, these possibilities requires experimental evidence.

Thank you for your comment highlighting an important point. We admit that our concept is somewhat speculative.
We have changed the title to "A gene regulatory network combining Pax3/7, Sox10 and Mitf generates diverse pigment cell-types in medaka and zebrafish".

2. The authors explained the mild xanthophore defects in pax3 mutants might result from partial compensation by pax7 at early stage. However, pax7 expression is reduced in pax3 mutants at early stage (Fig 4), which conflicts with their pax7 compensation hypothesis.

The reviewer is correct that the explanation for the mild xanthophore phenotype in *pax3* mutants is unclear. However, we note that *pax7*-expressing cells are retained in the anterior region, particularly in the head, in the *pax3* mutant (Fig. 4 D, H, J). We speculate, therefore, that *pax7* expression is not completely dependent on Pax3, and that residual Pax7, expressed independently of Pax3, can partially compensate for the loss of Pax3.
We have added this speculation in line 270 ‘....*pax7* expression is partially dependent on *pax3*’.

3. The conclusion pax7 serves as a cell fate switch that determines melanophore lineage or xanthophore/leucophore lineage is critical to the model they proposed. The ectopic expression of pax7a promoting xanthophore fate in the presence of mitfa is convincing. However, I think the cellular evidence is not sufficient. The phenotype of pax7a mutants could result from xanthophore-melanophore interactions or xanthophore-melanophore competition in the progenitor pool instead of genetic regulation and fate decision. Lineage tracing the cell differentiation of mitfa+ pax7-

© 2023. Published by The Company of Biologists under the terms of the Creative Commons Attribution License (https://creativecommons.org/licenses/by/4.0/).

cells vs. mitfa+ pax7+ cells would be a straightforward approach and necessary to support the model.

We agree that direct cellular evidence that Pax7 serves as a melanophore vs. xanthophore fate switch remains to be obtained, and other explanations are possible. We are planning the lineage tracing experiment and are preparing *mitfa*: RFP knockin medaka to cross with the *pax7a*: GFP knockins. However, this will take a long time, and we suggest it is not necessary in what is already a dense manuscript.

The reviewer suggests the possibility of a cell-cell interaction scenario as an alternative explanation.

Assuming that the phenotype of *pax7a* mutants (loss of xanthophores and increase in melanophores) results from cell-cell interaction or competition between xanthophores and melanophores, you would have to predict that the progenitor pool contains two distinct (independent) populations, *pax7a+* cells and *pax7a-* cells, and that *pax7a+* cells, which correspond to xanthophore progenitors, are lost in the *pax7a* mutant. Perhaps you would also have to predict that the *pax7a-* cells correspond to *mitfa+* cells because they differentiate into melanophores.
In this scenario, the loss of Mitf would result in an 'absence of melanophores' due to loss of *mitf+* cells and at the same time an 'increase in xanthophores' due to cell-cell interaction. However, this is not consistent with our results of the *mitf* mutant phenotype, which is rather the opposite in medaka, where xanthophores are completely lost. Therefore, the cell-cell interaction scenario does not seem compelling, and the most likely explanation for our results would be that the progenitor pool contains two populations, *pax7a+mitfa+* cells and *pax7a-mitfa+* cells.

4. I appreciate the authors' effort to investigate the mechanisms in parallel in medaka and zebrafish, which provides a broader picture to the audience. In the current manuscript, such comparative analysis indeed emphasizes the overall conservation of this network between teleosts. Yet an intriguing difference between the two species is that xanthophores are totally lost in medaka mitf mutant but not zebrafish mitf mutant. The authors didn't look further into this and only speculated that tfec might compensate for the loss of mitf in zebrafish. To address it by comparing tfec compensatory expression and function between medaka and zebrafish would strengthen the model, and promote the work to a better example of cross species comparative analysis.

Thank you for your nice suggestion. This is also what we find intriguing. In fact, we are currently preparing another substantial paper describing this point (phenotype due to double loss of Mitf and Tfec) for publication elsewhere. Our results support the speculation that the degree of redundancy of Mitf and Tfec in xanthophore development differs between medaka and zebrafish.

Minor issues:
1. The authors claim leucophores only exist in medaka but not zebrafish. In fact there are leucophores in adult zebrafish as well.

Thank you for your comment. The reviewer is, of course, correct that zebrafish have leucophores, but these have only been described from adult fish and are absent in the embryos that are the dominant focus of our paper, hence our simplification. In addition, zebrafish leucophores are of two types, only one of which may be homologous to those in medaka; their relationships in terms of evolutionary and/or developmental origin have yet to be assessed and may distinct. For simplicity, we had not mentioned these in the text. However, in response to the reviewer's comment, we have added a brief caveat to our description at lines 79-80, and referenced the relevant paper.

2. In Fig. 2A, arrows indicate 3 yellow cells and 1 white cell. The audience might be confused according to the description in the introduction, xanthophores are yellow and leucophores are white. In B, C and D, some arrows are pointing to blurry fluorescent cells. Could you find better examples to avoid the blurriness which would affect counting? There's no description for leucophores having fluorescent in the manuscript. In addition, there are similar ventral bright iridophores that could be mistaken as leucophores. I think additional descriptions would help readers to distinguish different cell types.

© 2023. Published by The Company of Biologists under the terms of the Creative Commons Attribution License (https://creativecommons.org/licenses/by/4.0/).

Thank you for your comment. We have added the descriptions of pigment cell characteristics in the Materials and Methods section in lines 608-614 as follows:

‘Note that larval leucophores appear orange to varying degrees and are clearly visible under darkfield illumination, whereas xanthophores appear yellowish but are ambiguous under brightfield illumination. Under UV light, leucophores show stronger autofluorescence than xanthophores, but iridophores and melanophores do not. Leucophores and xanthophores can be distinguished by their location: Leucophores are located along the dorsal and ventral midlines, whereas xanthophores are located laterally across the body surface in the larva.’

We have removed the arrows pointing to fluorescent leucophores in B and D because these images are intended to show xanthophores, which are not clearly visible under either brightfield or darkfield illumination (A, C).

These images are typical of wild-type and *pax3b* mutant, both of which normally have leucophores of different colors, from white to yellow or orange. It is easy to count leucophores as they are dull white to yellow/orange in darkfield and distinguishable from the other cell types (see also revised Suppl. Fig. 4).

Highly fluorescent cells in the ventral margin in Fig. 2B and in the dorsal margin in Fig. 2D are leucophores, not iridophores.

We have added these explanations in lines 851-854.

3. Suppl. Fig. 4 Leucophores are dispersed and distributed laterally in pax3b mutants compared to WT. Does pax3b mutation also affect leucophore morphology and patterning, or is it just individual variation? It’s hard to compare xanthophore, leucophore and iridophore in the close ups H, J and L.

The reviewer is correct in their observations.
The morphology of leucophores and others can be altered in situations or between individuals despite being wild-type or mutants.
As we previously reported (Nagao et al., 2014), it appears that leucophores are distributed more laterally when xanthophores are lost from the adjacent lateral region, indicating that cell-cell interaction with xanthophores seems to determine their relative position. This explanation has been added to the legend of Suppl. Fig. 4.
We have replaced the photos of whole larvae and adults with the new ones (all photos except for B, D, F) to make the above clearer. In particular, adult xanthophores, leucophores and iridophores are now clear enough to be observed with arrows and arrowheads pointing to leucophores and iridophores, respectively. We have revised the corresponding descriptions in the legend of Suppl. Fig. 4.

4. Suppl. Fig. 5 C, F and I look like green fluorescent channel rather than dark field. The example of pax3a mutants in D seems to have many more melanophores than WT, similarly in Suppl. Fig. 6B.

Thank you for your comment. The reason why the photos look like a green fluorescent channel is due to the white balance problem. We have adjusted the hue to make them look appropriate, without compromising scientific fairness.
The number of melanophores is variable (see quantitation in Suppl. Fig. 5 J), and may be slightly increased in the *pax3a* mutant shown in Suppl. Fig. 5, although it is not significantly different from that in WT.

5. Fig. 5 G-I in the figure legend but not in the figure.

Thank you for pointing out the mistake. We have corrected the sentence in line 896.

6. Fig. 6 There’s a white scale bar covered by the label gch.

We have deleted the scale bar (Fig. 7).

© 2023. Published by The Company of Biologists under the terms of the Creative Commons Attribution License (https://creativecommons.org/licenses/by/4.0/).

Second decision letter

MS ID#: DEVELOP/2023/202114

MS TITLE: A gene regulatory network combining Pax3/7, Sox10 and Mitf generates diverse pigment cell-types in medaka and zebrafish

AUTHORS: Motohiro Miyadai, Hiroyuki Takada, Akiko Shiraishi, Tetsuaki Kimura, Ikuko Watakabe, Hikaru Kobayashi, Yusuke Nagao, Kiyoshi Naruse, Shin-ichi Higashijima, Takashi Shimizu, Robert Kelsh, Masahiko Hibi, and Hisashi Hashimoto
ARTICLE TYPE: Research Article

I am happy to tell you that your manuscript has been accepted for publication in Development, pending our standard ethics checks.

Reviewer 1

Advance summary and potential significance to field

This paper illuminates a transcriptional regulatory network that governs lineage decisions within pigment cell precursors in teleost fish. It clarifies the roles of Pax3, Pax7 and Mitf in these decisions, and in particular presents evidence that Pax7 inhibits the ability of Mitf to promote the melanocyte lineage.

Comments for the author

My reservations from the first submission have been satisfactorily addressed.

Reviewer 2

Advance summary and potential significance to field

As before

Comments for the author

This revised manuscript deals well with the minor points I raised

Reviewer 3

Advance summary and potential significance to field

Deeper understanding of gene regulatory networks and their evolution underlying pigment patterns of teleosts.

Comments for the author

Authors have adequately addressed the concerns and suggestions.

© 2023. Published by The Company of Biologists under the terms of the Creative Commons Attribution License (https://creativecommons.org/licenses/by/4.0/).
